# E3 ubiquitin ligases orchestrate chemo-resistance in gastrointestinal malignancies: from DNA damage response to therapeutic targeting

**DOI:** 10.3389/fonc.2026.1835284

**Published:** 2026-08-27

**Authors:** Teng Ma, Yongzhu Zhou, Xiaojun Li, Junhong Guan

**Affiliations:** Cuiying Biomedical Research Center, Gansu Province Key Laboratory of Environmental Oncology, Lanzhou University Second Hospital, Lanzhou, Gansu, China

**Keywords:** cancer therapy resistance, cell cycle, DNA damage response, E3 ubiquitin ligase, gastrointestinal tumors, ubiquitination

## Abstract

Therapy resistance in gastrointestinal tumors remains a critical challenge in clinical oncology, severely compromising therapeutic efficacy and long-term patient survival. E3 ubiquitin ligases, as core enzymes involved in the protein ubiquitination process, have been demonstrated to play indispensable roles in regulating key biological processes in tumor cells, including DNA damage repair, cell cycle progression, apoptosis, cancer stem cell maintenance, and immune evasion. Additionally, E3 ligases are key regulators of resistance to multiple anticancer strategies, such as chemotherapy, radiotherapy, targeted therapy, and immunotherapy. This review systematically summarizes the functions and mechanisms of E3 ligases in DNA damage response and cancer therapy resistance, and further highlight recent advances in therapeutic approaches by targeting specific E3 ligases. Notably, combining E3 inhibitors with existing anticancer therapies may substantially enhance treatment responses and improve patient prognosis, providing a solid theoretical basis for future clinical applications.

## 1 Introduction

Gastrointestinal (GI) tumors represent one of the most prevalent and lethal malignancies worldwide, contributing to approximately one-third of all cancer-related mortality (1). Current therapeutic strategies for GI tumors include surgical resection, chemotherapy, radiotherapy, targeted therapy, and immunotherapy (2). Although treatment efficacy has improved markedly in recent decades, therapy resistance remains a critical challenge in clinical settings, particularly among patients with advanced or metastatic disease (3). Therapy resistance arises from multiple complex mechanisms, primarily including but not limited to tumor heterogeneity, physical barriers, DNA damage repair, growth dynamics, metabolic reprogramming, and the tumor microenvironment (3, 4). Understanding the specific molecular mechanisms conferring tumor resistance and developing novel strategies targeting these resistance biomarkers will significantly improve personalized therapy efficacy and benefit patients’ survival.

Genomic instability constitutes a hallmark of cancer (5). To preserve genome integrity following DNA damage or replication stress, cells deploy sophisticated surveillance mechanisms collectively termed the DNA damage response (DDR). This signaling network operates through a hierarchical cascade: sensors detect DNA lesions, transducers amplify and relay these signals to downstream effectors, which ultimately dictate cell fate decisions-–including cell cycle arrest, DNA repair, damage-induced transcriptional reprogramming, or programed cell death. The nature of DNA lesions determines the specific repair pathway engaged, encompassing direct reversal, mismatch repair (MMR), base excision repair (BER), nucleotide excision repair (NER), translesion synthesis (TLS), homologous recombination (HR), and non-homologous end joining (NHEJ). Notably, defective DDR signaling elevates cellular mutation rates, thereby promoting tumorigenesis; conversely, hyperactive DDR pathways confer resistance to chemotherapy and radiotherapy. Consequently, targeted modulation of DDR components represents a promising strategy to overcome therapeutic resistance (6, 7).

Post-translational modifications (PTMs) represent critical regulatory mechanisms governing DDR and therapeutic resistance (8). PTMs entail covalent chemical alterations of amino acid residues following protein synthesis, thereby modulating protein structure, function, and intermolecular interactions. Major PTM types include phosphorylation, acetylation, ubiquitination, SUMOylation, neddylation, glycosylation, and lactylation (9). Among these, ubiquitination, the enzymatic conjugation of ubiquitin to substrate lysines, profoundly influences cellular outcomes by regulating protein stability, localization, and signal transduction, and constitutes a pivotal driver of drug resistance (10, 11). This modification is mainly orchestrated by a three-tiered enzymatic cascade comprising ubiquitin-activating enzymes (E1), ubiquitin-conjugating enzymes (E2), and ubiquitin ligases (E3) (12).

**Figure 1:**
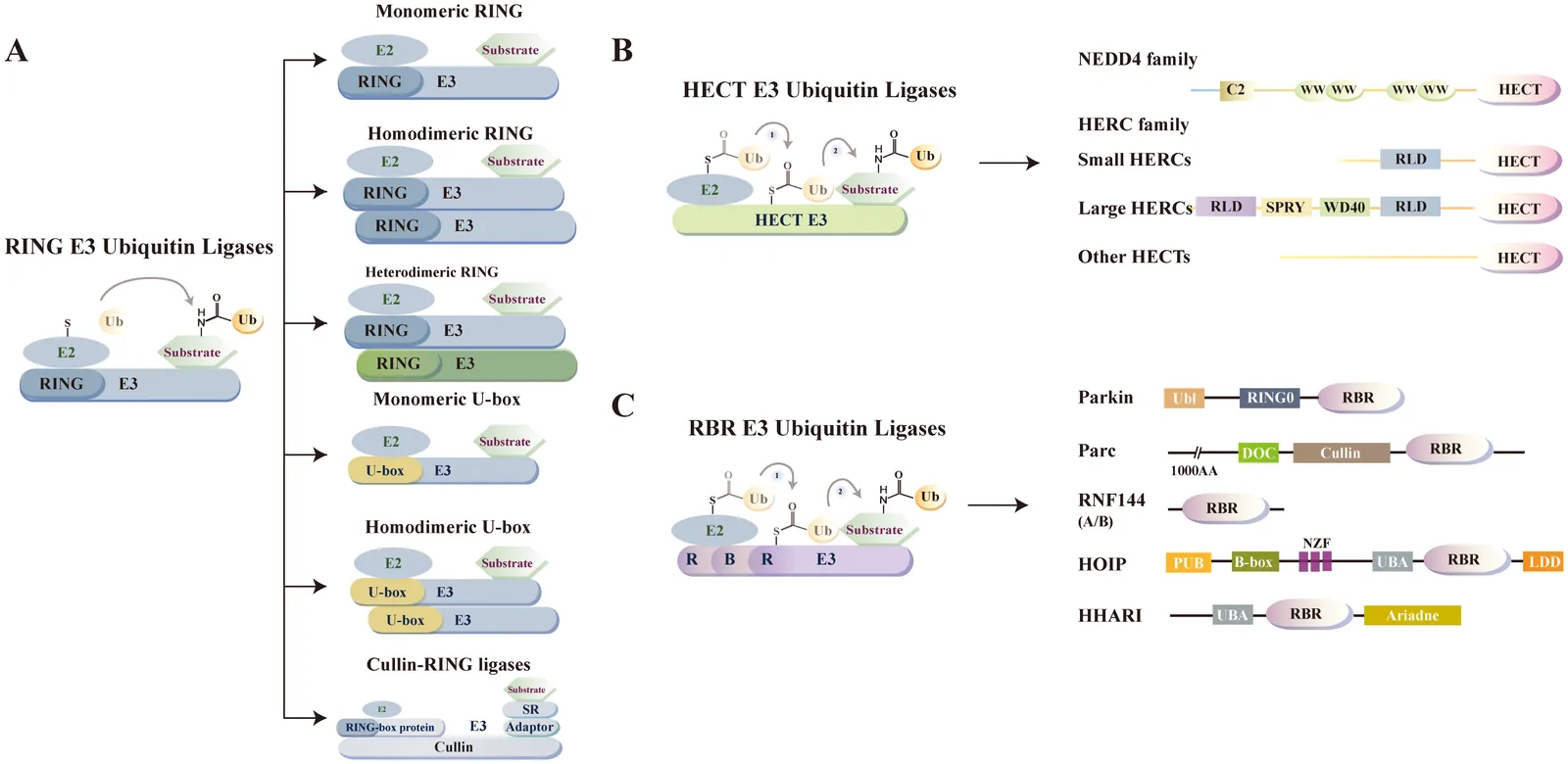
E3 ubiquitin ligase families and catalytic architectures. RING-type E3 ligases. **(A)** RING (Really Interesting New Gene) E3 ligases constitute the most abundant E3 class. They contain a RING finger or U-box domain that recruits E2 enzymes and facilitates direct ubiquitin transfer to substrates without covalent intermediate formation. RING E3s function as monomers, homodimers, or heterodimers. **(B)** HECT (Homologous to the E6-AP Carboxyl Terminus) E3 ligases possess a conserved C-terminal HECT domain with an active-site cysteine. HECT E3s employ a two-step mechanism: ubiquitin is first transferred from E2 to the HECT catalytic cysteine, forming a transient E3-ubiquitin thioester intermediate, then transferred to substrate lysines. Human HECT E3s are classified into three subfamilies based on N-terminal architecture: the NEDD4 family (WW domains), the HERC family (RCC1-like domains/RLDs), and other HECTs with diverse regulatory domains, conferring substrate specificity. **(C)** RBR (RING-in-between-RING) E3 ligases contain three zinc-binding domains: RING1, IBR, and RING2. They employ a hybrid RING-HECT mechanism: RING1 recruits the E2–ubiquitin conjugate, then ubiquitin is transferred to a RING2 catalytic cysteine, forming an E3-ubiquitin thioester intermediate before substrate conjugation. RBR members (Parkin, PARC/CUL9, RNF144A/B, HOIP/RNF31, HHARI/ARIH1) regulate protein quality control, DNA damage responses, immune signaling, and tumorigenesis through substrate-specific ubiquitination.

E3 ubiquitin ligases function as pivotal executors of the ubiquitin signaling cascade and are categorized into three distinct structural families: RING-type, HECT-type, and RBR-type (13) (**Figure 1**). By recognizing specific substrates and catalyzing ubiquitin transfer, E3 ligases confer substrate specificity and dictate the functional outcomes of ubiquitination, thereby occupying a central node in cellular regulatory networks governing DNA repair and cell cycle progression (14, 15). In the context of cancer, E3 ligases modulate DDR, tumorigenesis, and therapeutic resistance through the targeted ubiquitination and degradation of key oncogenic or tumor suppressor proteins (14). For instance, MDM2 overexpression promotes p53 degradation, thereby attenuating p53-mediated DNA damage responses and apoptosis while conferring drug resistance (16). Conversely, loss of VHL, a tumor suppressor E3 ligase, results in HIF-1α accumulation, angiogenic activation, and accelerated tumor progression (17). Furthermore, E3 ligase dysfunction is intimately linked to tumor microenvironment remodeling, immune evasion, and cancer stem cell maintenance, rendering these enzymes attractive targets for anti-cancer therapeutic strategies (15, 18).

In summary, E3 ubiquitin ligases serve as indispensable regulators of therapeutic resistance in cancer. Comprehensive characterization of their structural architectures, catalytic mechanisms, and regulatory networks not only illuminates the molecular underpinnings of treatment refractoriness but also establishes a robust framework for developing next-generation therapies to overcome resistance. With continued advances in the ubiquitin-proteasome system biology, precision targeting of E3 ligases has emerged as a transformative strategy to circumvent chemoresistance and radioresistance in gastrointestinal malignancies (18, 19).

**Figure 2:**
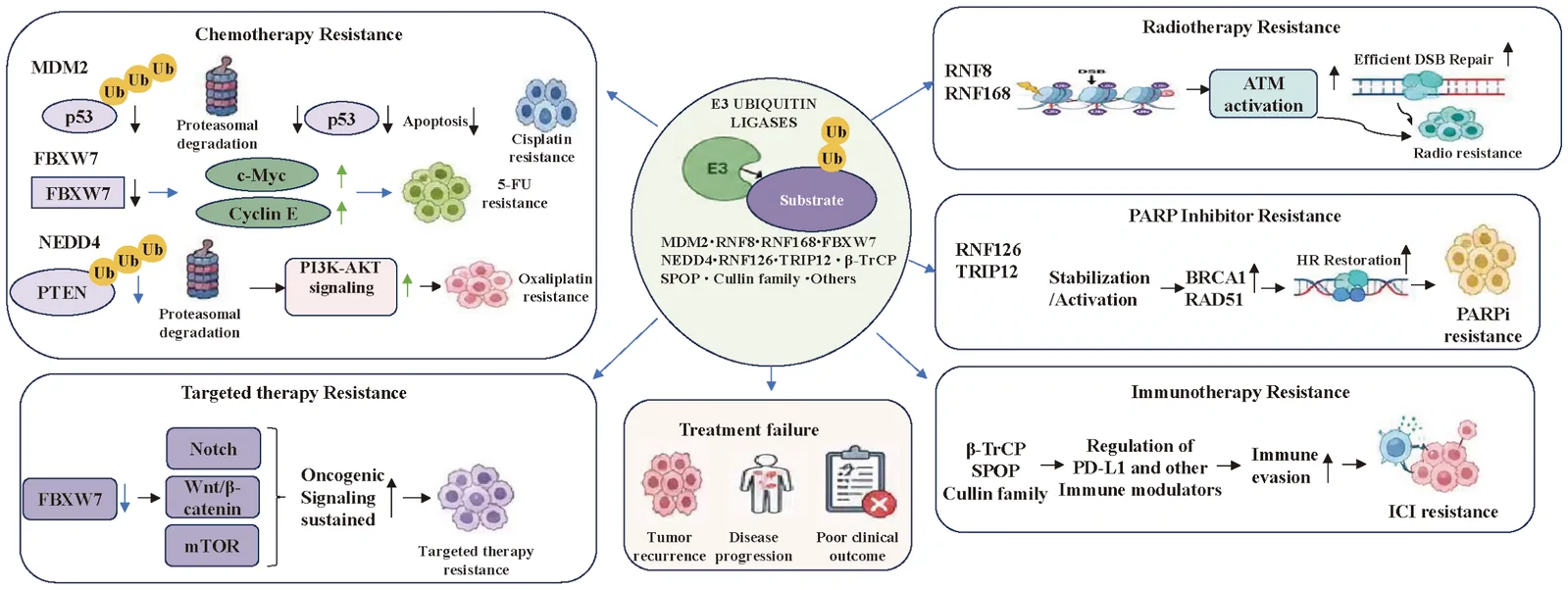
A unified mechanistic framework linking E3 ubiquitin ligases, DNA damage response (DDR) remodeling, cancer hallmarks, and therapy resistance in gastrointestinal tumors. Therapeutic stressors, including chemotherapy, radiotherapy, poly(ADP-ribose) polymerase inhibitors (PARPi), and oxidative stress, induce diverse forms of DNA damage, such as double-strand breaks (DSBs), single-strand breaks (SSBs), and replication stress. E3 ubiquitin ligases function as central regulatory hubs within the DDR network by modulating the stability, activity, and recruitment of key DDR components at the sensor (e.g., PARP1 and the MRN complex), transducer (ATM, ATR, DNA-PKcs, CHK1, and CHK2), and effector (BRCA1, RAD51, FANCD2, OGG1, and p53) levels. Distinct ubiquitin linkages exert different biological effects, with K48-linked ubiquitination primarily promoting proteasomal degradation and K63-linked ubiquitination facilitating signal transduction and repair-complex assembly. Through these mechanisms, E3 ligases remodel DDR signaling, enhance DNA repair capacity, regulate checkpoint activation, protect replication forks, and influence chromatin dynamics. Persistent DDR remodeling contributes to the acquisition of multiple cancer hallmarks, including adaptation to genomic instability, resistance to cell death, stemness and cellular plasticity, immune evasion, metabolic reprogramming, and autophagy/mitophagy. Collectively, these processes promote resistance to chemotherapy, radiotherapy, PARP inhibitors, targeted therapies, and immunotherapies in gastrointestinal malignancies.

To facilitate understanding of the central concepts discussed throughout this review, **Figure 2** presents a unified mechanistic framework illustrating how E3 ubiquitin ligases act as critical regulatory nodes linking DNA damage response (DDR) remodeling, cancer hallmark acquisition, and therapy resistance in gastrointestinal tumors. Upon genotoxic stress induced by chemotherapy, radiotherapy, or targeted therapies, E3 ligases dynamically regulate key DDR pathways, including DNA repair, checkpoint activation, and replication stress responses (20, 21). Dysregulation of these ubiquitin-dependent processes promotes hallmark-associated adaptive phenotypes, such as apoptosis resistance, cancer stemness, metabolic reprogramming, autophagy, and immune evasion, ultimately contributing to resistance to chemotherapy, PARP inhibitors, targeted therapies, and immunotherapy (22, 23). This framework provides the conceptual basis for understanding how aberrant E3 ligase signaling drives therapeutic resistance and highlights the potential of E3 ligases as actionable targets for overcoming treatment failure in gastrointestinal malignancies (24, 25).

### 1.1 A unified mechanistic framework linking E3 ubiquitination, DDR remodeling and hallmarks-associated therapy resistance

The DNA damage response (DDR) is not a linear pathway but a coordinated signaling network composed of damage sensors, signal transducers and downstream effectors that collectively determine cellular fate after genotoxic stress (26). In the context of cancer therapy, this network is continuously remodeled by ubiquitin-dependent proteostasis (27). E3 ubiquitin ligases, by determining substrate specificity and ubiquitin chain topology, act as central regulatory nodes that connect DNA damage signaling with therapeutic adaptation (28). We propose a unified mechanistic framework in which E3 ubiquitin ligases regulate therapy resistance at multiple hierarchical levels of the DDR network. At the sensor level, E3 ligases modulate proteins involved in DNA lesion recognition and repair initiation, including PARP1- and MRN-associated signaling (27). At the transducer level, E3-mediated ubiquitination regulates the intensity and duration of ATM-, ATR-, DNA-PKcs-, CHK1- and CHK2-dependent checkpoint activation (29). At the effector level, E3 ligases control key determinants of repair pathway choice and cell-fate decision-making, such as BRCA1, RAD51, FANCD2, OGG1 and p53 (29). The DDR is largely coordinated by the ATM–CHK2–p53 and ATR–CHK1 axes, with DNA-PKcs also functioning as a key apical kinase in DSB repair signaling (30). Activated p53 subsequently induces cell-cycle arrest, senescence or apoptosis through downstream targets including p21, PUMA and BAX (31). In contrast, replication stress generates RPA-coated single-stranded DNA that recruits ATR–ATRIP complexes, resulting in CHK1 activation (32). CHK1 suppresses CDC25 phosphatases and maintains S-phase and G2/M checkpoints, thereby facilitating DNA repair and replication fork stability (33). Importantly, E3 ubiquitin ligases extensively modulate both pathways. MDM2 controls p53 stability, RNF8 and RNF168 amplify ATM signaling through histone ubiquitination, whereas β-TrCP, TRIP12 and RNF126 influence checkpoint signaling and repair-pathway selection (27, 34–36).

Importantly, E3-mediated DDR remodeling is closely aligned with the Hallmarks of Cancer framework (37). By enhancing DNA repair capacity, E3 ligases promote adaptation to genomic instability; by degrading p53 or other pro-apoptotic substrates, they facilitate resistance to cell death; by modulating Wnt, Notch, Hippo, NF-κB and interferon-related pathways, they contribute to sustained proliferative signaling, phenotypic plasticity, stemness and immune evasion (22, 27, 38, 39). These hallmark-associated outputs ultimately converge on resistance to chemotherapy, radiotherapy, PARP inhibitors, targeted therapy and immunotherapy (40). Immune evasion represents another key hallmark modulated by E3 ubiquitin ligases in gastrointestinal cancers (39). E3 ligases regulate immune checkpoint molecules such as PD-L1 through ubiquitination, thereby influencing tumor-immune interactions and therapeutic resistance (41). For example, the E3 ligase β-TrCP mediates K48-linked ubiquitination and degradation of PD-L1, whereas other E3 ligases can stabilize PD-L1 via K63-linked ubiquitination or prevent its degradation through crosstalk with deubiquitinases (42, 43). Dysregulation of these processes enables tumor cells to evade cytotoxic T cell recognition and contributes to resistance to immune checkpoint blockade therapies (41). Integrating immune evasion into the DDR-Hallmarks framework underscores the multifaceted roles of E3 ligases in shaping therapy outcomes and provides potential avenues for combinatorial targeting strategies that couple DDR modulation with immunotherapy (44). Metabolic reprogramming is another hallmark modulated by E3 ubiquitin ligases, which enables cancer cells to adapt their energy and biosynthetic demands under therapeutic stress (12). NEDD4, for example, ubiquitinates PTEN, leading to its proteasomal degradation and consequent activation of the PI3K/AKT pathway (45). This promotes glycolysis, lipid synthesis, and overall anabolic metabolism, supporting cancer cell survival and therapy resistance (46). Through such ubiquitin-dependent metabolic regulation, E3 ligases link DDR signaling, cellular energy homeostasis, and hallmark-driven therapy resistance, highlighting potential targets for combinatorial therapeutic strategies that simultaneously modulate DDR, metabolism, and cell survival (47).

Beyond DNA repair and metabolic adaptation, E3 ubiquitin ligases also contribute to therapeutic resistance through regulation of autophagy and mitophagy (48). Autophagy serves as a critical stress-adaptation mechanism that enables cancer cells to survive under conditions of nutrient deprivation, oxidative stress, and therapy-induced damage (49). Among the best-characterized examples, the RBR-type E3 ligase Parkin mediates selective ubiquitination of damaged mitochondria and promotes mitophagy through recruitment of autophagic machinery (49). This process facilitates removal of dysfunctional mitochondria, limits reactive oxygen species accumulation, and preserves cellular fitness under therapeutic stress (50). In addition, several E3 ligases have been implicated in regulation of autophagy-related signaling pathways, including mTOR, AMPK, and Beclin-1-associated networks (48). By promoting cellular adaptation to genotoxic and metabolic stress, E3 ligase-mediated autophagy may enhance tumor cell survival following chemotherapy, radiotherapy, and targeted therapy (51). These findings suggest that autophagy represents an additional mechanism through which E3 ubiquitin ligases contribute to treatment resistance and tumor persistence (48).

In addition to regulating DNA repair and checkpoint signaling, E3 ubiquitin ligases contribute to therapy resistance through modulation of cancer stemness and phenotypic plasticity (52). Cancer stem cells (CSCs) are characterized by enhanced self-renewal capacity, tumor-initiating potential, and intrinsic resistance to chemotherapy and radiotherapy (53). Emerging evidence suggests that several E3 ligases regulate CSC maintenance by controlling key stemness-associated signaling pathways, including Notch, Wnt/β-catenin, Hedgehog, and c-Myc (54). FBXW7 provides a representative example of this mechanism. As a tumor suppressor E3 ligase, FBXW7 promotes degradation of multiple oncogenic substrates, including c-Myc, Cyclin E, and Notch intracellular domain (NICD) (55). Loss of FBXW7 results in aberrant activation of Notch and c-Myc signaling, thereby enhancing stem-cell-like properties, tumor plasticity, and therapeutic resistance (56). Similar stemness-promoting effects have also been reported for other E3 ligases through regulation of Wnt and Hippo signaling networks (57). These observations indicate that E3 ligases influence treatment outcomes not only through DDR remodeling but also through maintenance of CSC populations that survive therapeutic stress and drive disease recurrence (58). These mechanistic insights are primarily derived from *in vitro* studies using colorectal cancer cell lines, with limited validation in xenograft models (59). Clinical evidence remains sparse, and variability in experimental conditions may affect reproducibility (60).

An important yet often overlooked aspect of E3 ligase biology is ubiquitin chain specificity. Different ubiquitin linkages generate distinct biological outcomes and therefore determine how E3 ligases influence DDR signaling (61). K48-linked polyubiquitination typically targets substrates for proteasomal degradation, whereas K63-linked polyubiquitination primarily functions as a non-proteolytic signaling platform that facilitates protein recruitment and signal propagation (62). In the DDR network, these distinct ubiquitin codes play fundamentally different roles. For example, MDM2 predominantly catalyzes K48-linked ubiquitination of p53, leading to its degradation and suppression of apoptosis (63). In contrast, RNF8 and RNF168 mainly promote K63-linked ubiquitination of histones at DNA damage sites, creating docking platforms for downstream repair factors such as 53BP1 and BRCA1 (64). Consequently, E3 ligases can regulate therapy resistance not only through substrate degradation but also through non-proteolytic signaling mechanisms that orchestrate DNA repair and checkpoint activation. Therefore, understanding ubiquitin chain topology is essential for interpreting the diverse functions of E3 ligases in gastrointestinal cancers and may provide additional opportunities for selective therapeutic intervention (22).

Non-proteolytic ubiquitination plays a critical role in DDR signaling beyond substrate degradation. K63-linked ubiquitin chains, mediated by E3 ligases such as RNF8 and RNF168, serve as scaffolds for the recruitment of downstream repair factors, including 53BP1 and BRCA1, at sites of DNA damage (65). This non-degradative modification facilitates signal amplification and efficient repair complex assembly, distinguishing it mechanistically from K48-linked ubiquitination that targets proteins like p53 for proteasomal degradation (23). By orchestrating DNA repair and checkpoint signaling through non-proteolytic ubiquitination, E3 ligases contribute to therapy resistance in a manner that is independent of protein turnover (66). Understanding these signaling functions is essential for interpreting DDR remodeling and designing E3-targeted therapeutic strategies (67).

Crosstalk between ubiquitination and other post-translational modifications (PTMs) adds an additional layer of regulatory complexity in DDR signaling (66, 68). SUMOylation and phosphorylation can prime substrates for ubiquitination or modulate E3 ligase activity, thereby influencing DNA repair efficiency and therapy response (68). A notable example is RNF4, a SUMO-targeted ubiquitin ligase (STUbL), which recognizes SUMOylated substrates and mediates their ubiquitination to facilitate DNA damage repair complex assembly (69). Similarly, phosphorylation of DDR components can regulate their recognition by specific E3 ligases, coordinating repair, checkpoint activation, and apoptosis (66). Integrating these cross-PTM interactions into DDR networks highlights the multifaceted roles of E3 ligases in orchestrating therapy resistance and underscores the importance of considering combinatorial PTM regulation in therapeutic targeting strategies (68).

Beyond canonical DNA repair pathways, E3 ubiquitin ligases play essential roles in replication stress responses and chromatin remodeling, both of which are increasingly recognized as major determinants of therapeutic resistance (66). Replication stress, characterized by stalled or collapsed replication forks, represents a common consequence of oncogene activation and exposure to DNA-damaging therapies (70). In response, ATR–CHK1 signaling coordinates fork stabilization and replication recovery, while multiple E3 ligases regulate the abundance and activity of proteins involved in replication fork protection and restart (71). Chromatin remodeling constitutes another critical layer of DDR regulation. Because DNA lesions occur within highly organized chromatin structures, repair factors must gain access to damaged DNA through coordinated chromatin modifications (65). Among the best-characterized examples, RNF8 and RNF168 catalyze ubiquitination of histones surrounding DNA damage sites, generating signaling platforms that recruit downstream repair proteins such as 53BP1 and BRCA1 (65). These ubiquitin-dependent chromatin alterations facilitate damage signaling, repair-pathway selection, and maintenance of genome stability (65). Consequently, dysregulation of E3 ligase-mediated chromatin remodeling may enhance DNA repair capacity and contribute to resistance against chemotherapy, radiotherapy, and PARP inhibitors (65, 72).

Thus, E3 ubiquitin ligases should not be viewed merely as isolated regulators of individual substrates. Instead, they function as systems-level coordinators that couple ubiquitination, DDR network rewiring and cancer hallmark acquisition. This framework provides a conceptual basis for understanding how diverse E3 ligases converge on common drug-resistance phenotypes in gastrointestinal malignancies and supports rational development of E3-targeted combination therapies.

Unlike previous reviews summarizing the ubiquitin-proteasome system across multiple cancer types (22), this review specifically focuses on gastrointestinal malignancies and systematically integrates E3 ligase-mediated regulation of DNA damage response with therapy resistance, highlighting both mechanistic insights and therapeutic strategies. To facilitate comparison among the major E3 ubiquitin ligases discussed throughout this review, the representative E3–substrate pairs, associated DDR pathways, and therapy-resistance phenotypes are summarized in **Table 1**.

**Table 1:** E3 ubiquitin ligases linking DNA damage response remodeling to therapy resistance.

E3 ubiquitin ligase	Major substrate(s)	DDR pathway / biological process	Impact on therapy resistance	Representative cancer type
MDM2	p53	ATM–CHK2–p53 signaling	Suppresses apoptosis and promotes chemoresistance	Gastric cancer, CRC
RNF8	Histones (H2A/H2AX), repair proteins	DNA double-strand break signaling	Facilitates DNA repair and radioresistance	GI cancers
RNF168	53BP1-associated repair machinery	DSB repair pathway	Enhances genome maintenance and treatment tolerance	GI cancers
RNF4	SUMOylated DDR proteins	DNA damage signaling regulation	Supports cellular adaptation to genotoxic stress	GI cancers
RNF126	E2F1/BRCA1 axis	Homologous recombination (HR)	Promotes HR repair and PARP inhibitor resistance	Breast and GI cancers
TRIP12	PARP1	PARP inhibitor response pathway	Restricts PARP1 trapping and reduces PARPi efficacy	Multiple solid tumors
NEDD4-1	PTEN	PI3K/AKT survival signaling	Enhances survival signaling and drug resistance	Colorectal cancer
ARIH2	p21	Cell-cycle checkpoint regulation	Reduces chemosensitivity through p21 degradation	Gastric cancer
Parkin (PARK2)	Microtubule-associated proteins	Mitophagy and stress adaptation	Modulates paclitaxel sensitivity	Gastrointestinal and breast cancers
HUWE1	TOMM20	Mitochondrial homeostasis	Increases oxaliplatin sensitivity when activated	Colorectal cancer
TRIM25	Caspase-7-associated pathway	Apoptotic signaling	Impairs apoptosis and promotes resistance	Colorectal cancer
ARIH1	PHB1	Oxidative phosphorylation regulation	Supports tumor progression and metabolic adaptation	Colorectal cancer

## 2 Structure and function of E3 ligases

### 2.1 RING-type E3 ligases

RING-type E3 ligases serve as core catalytic factors in the ubiquitin-proteasome system (UPS), playing pivotal roles in protein degradation and signal regulation (73). The RING (Really Interesting New Gene) domain was originally identified in 1991 within a lymphoma-associated gene product by Freemont and colleagues (74). Subsequent studies revealed the widespread distribution of RING domains across diverse protein families and their intimate association with ubiquitination cascades (75). Structurally, RING domains are characterized by zinc-rich coordination centers where adjacent zinc ions stabilize “zinc finger” configurations through coordinating residues, thereby maintaining protein conformation and catalytic integrity, providing the structural foundation for function within complex cellular environments (73).

As the largest E3 ligase class within the UPS, RING-type enzymes are defined by the presence of either a canonical RING domain (zinc-binding) or a structurally analogous zinc-free U-box domain. These domains specifically recruit E2 ubiquitin-conjugating enzymes and catalyze ubiquitin transfer from E2 to substrates, thereby governing cellular homeostasis, subcellular localization, and protein function (76). Functionally, RING-type E3s operate through diverse architectures: they may function as monomers (e.g., MDM2, CBL), homodimers (e.g., RNF4), or heterodimers (e.g., BRCA1/BARD1). Notably, homodimers can simultaneously bind two E2 enzymes to enhance ubiquitination efficiency, whereas heterodimers typically interact with a single E2 and exhibit distinct substrate specificity (**Figure 1A**) (77, 78).

Beyond these simple forms, multi-subunit Cullin–RING ligases (CRLs) represent a major expansion of RING-type functional diversity. Employing Cullin as a molecular scaffold, CRLs recruit substrate receptors (e.g., F-box proteins, SOCS box proteins) via their C-terminal regions while binding RING proteins (RBX1/2) at the N-terminus, thereby assembling diverse ubiquitination pathways such as SCF and CRL2-VHL complexes (79). Similarly, the anaphase-promoting complex/cyclosome (APC/C), a 19-subunit RING-type E3 comprising Apc11 and Apc2, mediates the degradation of cell cycle regulators including Cyclin B, thereby orchestrating cell cycle progression (80).

Throughout the ubiquitination cascade, RING-type E3s primarily function as molecular scaffolds that facilitate direct ubiquitin transfer from E2 enzymes to substrates. Their RING domains specifically engage E2 enzymes, spatially orienting the E2~Ub thioester in precise proximity to substrate lysine ϵ-amino groups, thereby markedly enhancing ubiquitin transfer kinetics (81). Unlike HECT-type E3s that form obligatory ubiquitin-thioester intermediates, RING-type E3s operate through non-covalent catalysis, accelerating E2-mediated ubiquitin discharge by stabilizing the catalytically competent E2~Ub conformation and lowering the activation energy barrier (82). This catalytic strategy confers superior reaction rates and substrate selectivity, enabling rapid adaptation to dynamic cellular signals and robust maintenance of homeostasis. RING-type E3 activity is subject to sophisticated regulation through conformational dynamics, regulatory factor interactions, and sensing of redox states and ionic microenvironments (83). This multi-layered control ensures signal fidelity, preventing spurious ubiquitination events that could disrupt cellular homeostasis. In gastrointestinal malignancies, RING-type E3s critically modulate the proliferation-apoptosis equilibrium by targeting tumor-associated proteins for proteasomal degradation, thereby influencing malignant progression and therapeutic resistance (84–86).

Among RING-type E3s, MDM2 represents a paradigmatic regulator. Through its RING domain, MDM2 recruits E2 enzymes to catalyze p53 and Bax ubiquitination, consequently upregulating Bcl-2 expression. This cascade suppresses p53-mediated cell cycle arrest and apoptotic signaling, fostering tumor cell survival and proliferation (87). Aberrant MDM2 overexpression further promotes chemoresistance through dual mechanisms: p53 inactivation attenuates DNA damage-induced cell cycle arrest while enhancing DNA repair capacity (88); concomitantly, MDM2 drives epithelial-mesenchymal transition (EMT), augmenting tumor cell motility, invasive capacity, and therapeutic tolerance (89).

Similarly, c-Cbl governs signal transduction kinetics by targeting receptor tyrosine kinases (RTKs) for ubiquitin-mediated degradation and modulating endocytic trafficking of receptors such as EGFR (90). In gastrointestinal tumors, c-Cbl abnormalities lead to prolonged activation of related pathways, promoting cell proliferation and altering chemotherapy sensitivity (91). Distinctly, RNF43 suppresses Wnt pathway hyperactivation by promoting ubiquitination and lysosomal clearance of Wnt receptor complexes (92, 93). These E3 ligases constitute complementary regulatory networks converging on the p53, RTK, and Wnt axes to orchestrate gastrointestinal tumor pathophysiology.

Collectively, representative RING-type E3 ligases precisely govern the stability and functional states of tumor-associated proteins through specific ubiquitin modifications, playing indispensable roles in gastrointestinal tumorigenesis and therapeutic response. However, current understanding of multi-pathway synergistic networks and spatiotemporal signaling control remains incomplete. Elucidating their precise mechanisms within complex pathological contexts may establish a robust theoretical framework for developing next-generation targeted therapeutic strategies.

### 2.2 HECT-type E3 ligase

HECT-type E3 ligases constitute a distinct class of ubiquitin-proteasome system enzymes characterized by unique C-terminal catalytic domains. The HECT (Homologous to the E6-AP Carboxyl Terminus) domain comprises approximately 350 amino acid residues and contains a highly conserved catalytic cysteine essential for ubiquitin transfer (94). Mechanistically, HECT-type E3s execute a two-step catalytic process: they first accept ubiquitin from E2 enzymes via a reversible thioester intermediate formed with their active site cysteine, subsequently transferring ubiquitin to substrate lysine (**Figure 1B**) (95). Substrate recognition is mediated primarily by N-terminal domains, which classify human HECT E3s into three subfamilies: the Nedd4 family (containing WW motifs), the HERC family (harboring RCC1-like domains, RLDs), and a heterogeneous group of “other” HECTs with diverse domain architectures (**Figure 1B**) (96). HECT E3 catalytic activity is maintained in an autoinhibited state through intramolecular interactions, with activation occurring upon specific signaling stimuli. Additionally, HECT-type E3s are subject to extensive post-translational regulation including phosphorylation, ubiquitination, and SUMOylation, which modulate enzyme conformation and substrate binding affinity, thereby fine-tuning ubiquitination efficiency (97).

HECT-type E3 ligases play pivotal roles in oncogenesis, tumor progression, and therapeutic resistance (75). In gastrointestinal malignancies, they orchestrate chemoresistance through four interconnected mechanisms governing cell survival, drug efflux, DNA repair, and stemness maintenance (98). These effects are mediated through four major mechanisms: (1) SMURF1/2-mediated PTEN degradation activates PI3K/AKT signaling, while NEDD4–1 cooperates to negatively regulate p53, collectively suppressing chemotherapy-induced apoptosis and enhancing tumor cell viability (99). (2) E6AP/UBE3A stabilizes P-glycoprotein (ABCB1/MDR1), attenuating chemotherapeutic cytotoxicity. Concurrently, NEDD4–1 and WWP2 enhance MRP1 (ABCC1)- and BCRP (ABCG2)-mediated efflux of platinum compounds and irinotecan, diminishing intracellular drug accumulation and promoting resistance (100). (3) HERC2 stabilizes BRCA1 to augment homologous recombination (HR) capacity, counteracting PARP inhibitor-induced synthetic lethality. Additionally, UBE3C and UBR5 amplify FANCD2-mediated Fanconi anemia pathway activity and Rad51-dependent double-strand break repair, driving resistance to cisplatin and radiotherapy (101). (4) NEDD4 and WWP1 sustain cancer stem cell populations via Notch and Wnt/β-catenin signaling, whereas ITCH enhances self-renewal through Hippo-YAP pathway modulation (102). These mechanisms collectively elevate recurrence risk and compromise patient survival. Furthermore, HECT-type E3-mediated ubiquitination regulates tumor microenvironment remodeling, indirectly modulating chemotherapy efficacy (103). Consequently, targeting HECT-type E3 ligases represents a promising therapeutic strategy to overcome chemo-resistance in gastrointestinal malignancies.

### 2.3 RBR-type E3 ligase

RBR-type E3 ubiquitin ligases (RING-in-between-RING) constitute a distinct E3 family characterized by a tripartite zinc-binding architecture comprising RING1, IBR (in-between-RING), and RING2 domains (104). Unlike canonical RING-type or HECT-type ligases, RBR E3s employ a hybrid catalytic mechanism: they first accept ubiquitin from E2 enzymes via a thioester intermediate formed with their active site cysteine, subsequently transferring ubiquitin to substrate lysines (**Figure 1C**) (104, 105). This RING-HECT hybrid mechanism confers exceptional specificity and regulatory complexity upon ubiquitin transfer reactions.

The human RBR family encompasses 14 members, including ARIH1, ARIH2, RNF14, RNF31, RNF144B, RNF216, and RBCK1 (106). These E3 ligases exert context-dependent, bidirectional regulatory effects during tumorigenesis, tumor progression, and therapeutic resistance (104): ARIH1 promotes cisplatin resistance in colorectal cancer through coordinated destabilization of p53 and NF2 (107); RNF31 and RBCK1 synergistically activate TNF-α/NF-κB signaling, establishing an anti-apoptotic barrier that attenuates chemotherapy-induced cell death (108); RNF144B modulates DNA damage responses and cell cycle progression through both p53-dependent and p53-independent mechanisms (109). Notably, while RNF216 and RBCK1 have been implicated in chemo-resistance (104), their precise molecular mechanisms remain elusive. Conversely, ARIH2, PARK2, and PARC maintain genomic integrity by stabilizing p53 and other core regulators (110); ARIH2 deficiency impairs DNA repair capacity, fostering tumor cell survival and chemotherapeutic evasion (111); additionally, PARC modulates drug sensitivity through p53-independent pathways (112).

Collectively, RBR-type E3 ligases orchestrate gastrointestinal tumor initiation, progression, and drug resistance through multifaceted mechanisms encompassing substrate recognition, ubiquitin chain topology determination, and hybrid catalysis. By governing the degradation of oncoproteins and tumor suppressors, RBR E3s critically influence tumor cell proliferation, metastatic potential, and therapeutic responsiveness, establishing these enzymes as pivotal targets for cancer biology research and therapeutic development (113, 114).

## 3 E3 ligases regulate chemotherapy resistance

### 3.1 Cisplatin resistance

Cisplatin, the most widely employed platinum-based chemotherapeutic agent, exerts cytotoxicity by forming DNA adducts that trigger genotoxic stress and apoptotic cell death (115). However, tumor cells frequently acquire cisplatin resistance through enhanced DDR capabilities, particularly via modulation of DDR-associated protein stability and activity (116). E3 ubiquitin ligases, as critical regulators of protein ubiquitination, specifically recognize and modify DDR-related substrates, thereby governing their degradation, functional status, and ultimately cellular cisplatin sensitivity (11).

**Figure 3:**
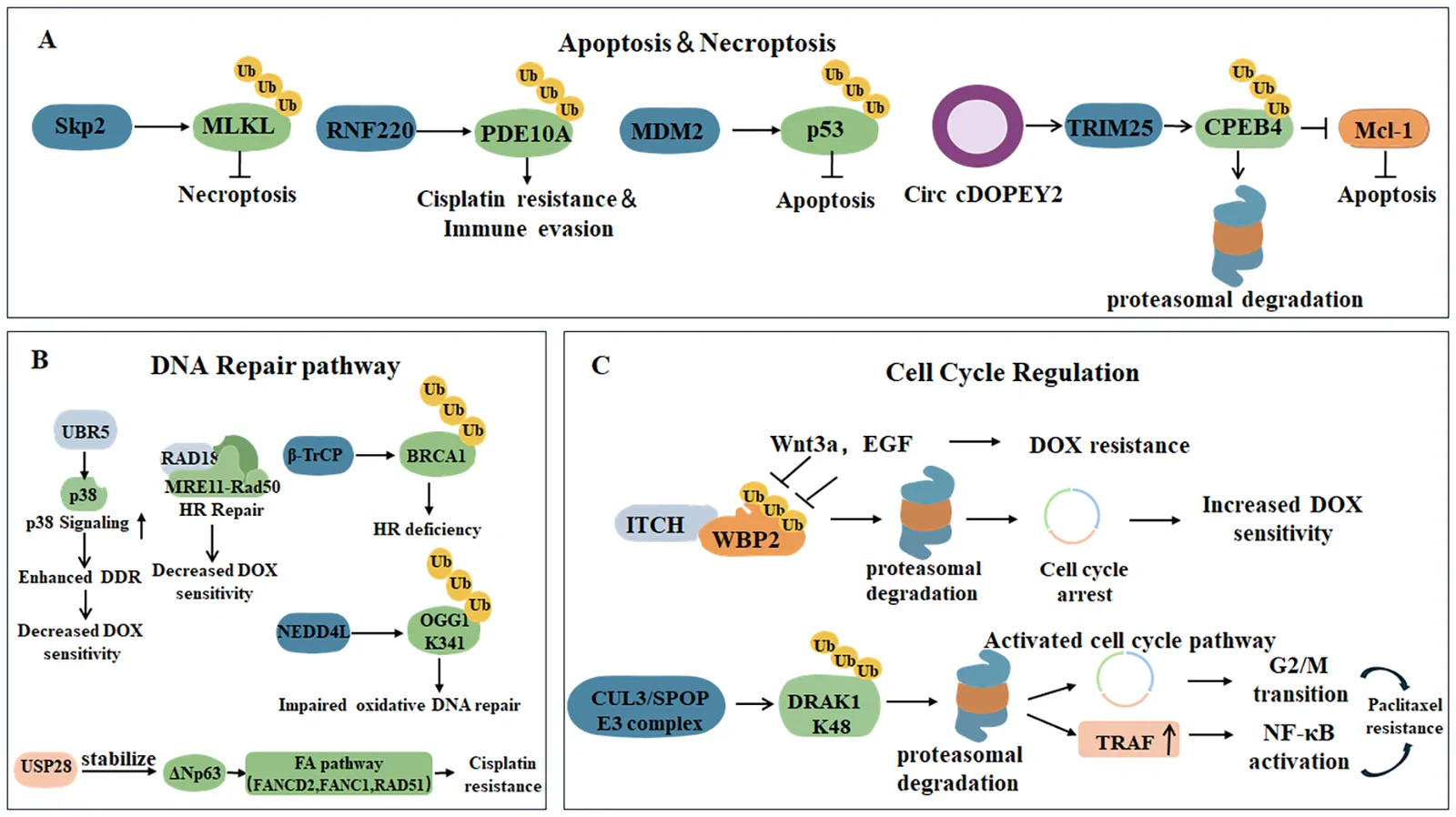
E3 ubiquitin ligases modulate chemotherapeutic sensitivity through cell death, DNA damage response and cell cycle control. **(A)** Apoptosis and necroptosis regulation. Skp2-MLKL degradation attenuates necroptosis and reduces cisplatin sensitivity. RNF220-directed PDE10A ubiquitination and degradation promotes cisplatin resistance and immune evasion. MDM2 drives p53 ubiquitination and degradation, suppressing p53-dependent apoptosis and conferring cisplatin resistance. Circular RNA cDOPEY2 enhances TRIM25-mediated CPEB4 degradation, downregulating anti-apoptotic Mcl-1 and increasing cisplatin sensitivity. **(B)** DDR pathway modulation. UBR5 overexpression activates p38 signaling to enhance DDR and attenuate doxorubicin-induced apoptosis. RAD18 interacts with MRE11 to promote MRN complex assembly and homologous recombination repair, augmenting DNA damage tolerance and doxorubicin resistance. β-TrCP ubiquitinates BRCA1 to promote its degradation, impairing HR repair and enhancing cisplatin sensitivity. NEDD4L ubiquitinates OGG1 at lysine 341, regulating its stability and base excision repair activity against oxidative DNA lesions (e.g., 8-oxoguanine). Additionally, the deubiquitinating enzyme USP28 stabilizes ΔNp63 to upregulate Fanconi anemia pathway genes (FANCD2, FANCI, RAD51C), enhancing DNA repair capacity and cisplatin resistance. **(C)** Cell cycle control. ITCH-mediated WBP2 ubiquitination and degradation induces cell cycle arrest and increased DOX sensitivity; however, Wnt3a and EGF signaling disrupt ITCH-WBP2 interaction, stabilizing WBP2 to activate cell cycle cascades and promote doxorubicin resistance. Additionally, the CUL3/SPOP complex recognizes DRAK1 and mediates its K48-linked polyubiquitination and degradation. DRAK1 loss compromises G2/M checkpoint integrity, enabling rapid recovery from paclitaxel-induced arrest and mitotic re-entry, thereby conferring resistance. DRAK1 degradation further upregulates TRAF6, activating NF-κB signaling to promote survival and exacerbate paclitaxel resistance.

Multiple E3 ligases modulate cisplatin response through distinct mechanisms. MDM2 promotes p53 ubiquitination and degradation, suppressing p53-mediated apoptosis and conferring cisplatin resistance in gastric cancer (**Figure 3A**) (117). Conversely, the HECT-type E3 HACE1 ubiquitinates Cyclin C to promote mitochondrial fission and ROS production, thereby sensitizing cells to cisplatin-induced apoptosis; HACE1 loss or Cyclin C ubiquitination inhibition markedly reduces cisplatin sensitivity (118). In colorectal cancer, Skp2-mediated ubiquitination and degradation of the necroptosis regulator MLKL attenuates cisplatin cytotoxicity, while Skp2 inhibition restores MLKL expression and enhances therapeutic efficacy (**Figure 3A**) (119). Additionally, in hepatocellular carcinoma, RNF220 ubiquitinates and degrades phosphodiesterase 10A (PDE10A), promoting both cisplatin resistance and immune evasion (**Figure 3A**) (120). These findings illustrate the dual capacity of E3 ligases to regulate DNA damage responses and immune modulation.

E3 ligases critically influence cisplatin sensitivity through direct regulation of DNA repair protein stability. β-TrCP promotes BRCA1 ubiquitination and degradation, impairing homologous recombination (HR) repair and increasing cisplatin sensitivity; conversely, β-TrCP deficiency stabilizes BRCA1, enhances HR capacity, and drives resistance (**Figure 3B**) (121). NEDD4 family HECT-type E3s maintain genomic stability through multi-substrate regulation of DDR pathways (122). Specifically, NEDD4L recognizes OGG1 lysine 341 and mediates its ubiquitination, thereby modulating OGG1 activity in base excision repair (BER) of oxidative DNA damage (8-oxoG) (123). NEDD4L-deficient cells accumulate OGG1, exhibit heightened DDR, and display marked cisplatin resistance (**Figure 3B**) (124).

Beyond direct DDR protein modification, E3 ligases regulate cisplatin resistance through alternative signaling pathways. USP28 stabilizes the transcription factor ΔNp63, thereby upregulating Fanconi anemia pathway genes (FANCD2, FANCI, RAD51C) to enhance DNA repair capacity and promote resistance (**Figure 3B**) (125). UBR5 activates p38/NF-κB signaling in ovarian cancer, conferring resistance to cisplatin and doxorubicin (**Figure 3B**) (126). Furthermore, non-coding RNAs modulate E3 ligase activity to influence drug sensitivity: circular RNA cDOPEY2 enhances TRIM25-mediated CPEB4 ubiquitination and degradation in esophageal squamous cell carcinoma, suppressing anti-apoptotic Mcl-1 expression and sensitizing cells to cisplatin (**Figure 3A**) (127). Long non-coding RNA SCAT7 regulates ubiquitination of RNA-binding proteins and DNA topoisomerase I to maintain DNA repair function and influence cisplatin resistance (128).

Collectively, E3 ubiquitin ligases orchestrate cisplatin resistance by modulating DNA damage signaling, apoptotic pathways, and alternative regulatory networks through substrate-specific ubiquitination. Elucidating the mechanistic contributions of distinct E3 ligase-substrate pairs to cisplatin resistance will establish a robust foundation for developing targeted therapeutic strategies to overcome cisplatin refractoriness.

### 3.2 Doxorubicin resistance

Doxorubicin (DOX), a cornerstone anthracycline chemotherapeutic agent widely employed in gastrointestinal malignancies, faces significant clinical limitations due to acquired tumor resistance (129). E3 ubiquitin ligases critically modulate cell cycle dynamics and apoptotic responses, thereby governing tumor cell sensitivity to DOX. While DOX exerts cytotoxicity through DNA damage induction and cell cycle arrest, tumor cells frequently evade these effects by manipulating E3-mediated protein degradation to alter cyclin levels and associated regulatory factors (130).

Recent evidence suggests that the E3 ubiquitin ligase UBR5 is overexpressed in triple-negative breast cancer, where it modulates the DDR via the p38 signaling pathway to resist DOX-induced cell death. Knockout of UBR5 impairs ATM-CHK2 signaling and cell cycle arrest, leading to accumulation of DNA damage and increased cellular sensitivity to DOX (**Figure 3B**) (131). Another E3 ubiquitin ligase RAD18 interacts with MRE11 to enhance MRN complex assembly and HR repair activation in osteosarcoma cells, thereby augmenting DNA damage tolerance and DOX resistance (**Figure 3B**). RAD18 inhibition restores DOX sensitivity, demonstrating the therapeutic potential of targeting E3-mediated repair mechanisms (132).

Additionally, the HECT-type E3 ITCH regulates DOX sensitivity through WBP2 degradation. ITCH recognizes the PPxY motif of WBP2 via its WW domain, mediating WBP2 ubiquitination and proteasomal elimination. ITCH loss-of-function mutations exacerbate resistance, whereas ITCH re-expression reverses WBP2-mediated chemoprotection (133). Wnt3a and EGF signaling can competitively bind the PPxY motif of WBP2, inhibiting ITCH-WBP2 interaction. This reduces WBP2 ubiquitination, increases its stability, activates cell cycle-related signaling pathways, and ultimately leads to DOX resistance (**Figure 3C**) (134).

Collectively, E3 ubiquitin ligases orchestrate DOX resistance through multifaceted regulation of cell cycle machinery, DNA repair capacity, and signal transduction pathways. Targeting specific E3 ligases or modulating their substrate degradation represents a promising strategy to overcome DOX refractoriness in tumors.

### 3.3 Paclitaxel resistance

Paclitaxel, a cornerstone microtubule-stabilizing agent widely employed in gastrointestinal cancer chemotherapy, exerts antimitotic effects by suppressing microtubule dynamics, thereby inducing mitotic arrest and apoptotic cell death (135). Emerging evidence indicates that E3 ubiquitin ligases govern tumor cell sensitivity to paclitaxel through targeted degradation of cell cycle and mitotic regulatory proteins (136, 137).

E3 ligases enable paclitaxel resistance by promoting checkpoint recovery and mitotic progression. The CUL3/SPOP complex specifically recognizes the cell cycle checkpoint kinase DRAK1 and mediates its K48-linked polyubiquitination and proteasomal degradation. DRAK1 loss compromises G2/M checkpoint integrity, permitting rapid cell cycle re-entry and paclitaxel resistance (138). Additionally, DRAK1 degradation upregulates TRAF6, activating NF-κB signaling to enhance tumor cell survival and proliferation, further exacerbating resistance (**Figure 3C**) (139).

E3 ligases additionally modulate microtubule-associated protein stability, thereby altering tubulin polymerization dynamics. While paclitaxel stabilizes microtubules to block mitosis, E3-mediated degradation of tubulin-regulatory proteins shifts the polymerization-depolymerization equilibrium, attenuating paclitaxel’s cytoskeletal interference and fostering resistance (140). Through this mechanism, tumor cells can adjust microtubule stability to attenuate paclitaxel’s effects and develop resistance.

Notably, E3 ligase-mediated ubiquitination processes not only influence cell mitosis but are also closely associated with oxidative stress responses. Paclitaxel treatment elevates intracellular ROS levels, promoting oxidative stress and apoptosis. However, specific E3 ligases mitigate ROS accumulation by targeting antioxidant proteins for ubiquitin-mediated degradation, thereby diminishing paclitaxel cytotoxicity and promoting chemoresistance (141). By targeting these ligases, tumor cells would can increase ROS accumulation and can enhance paclitaxel-induced cell death, thus overcoming resistance.

Collectively, E3 ubiquitin ligases orchestrate paclitaxel resistance through convergent mechanisms encompassing checkpoint bypass, microtubule remodeling, and oxidative stress adaptation. These multifaceted actions enable tumor cells to evade paclitaxel-induced mitotic catastrophe, establishing E3 ligases as critical targets for overcoming therapeutic refractoriness.

## 4 E3 ligases regulate PARP inhibitor resistance

PARP inhibitors (PARPis) represent a paradigm of synthetic lethality-based cancer therapy, particularly effective against homologous recombination-deficient (HRD) tumors harboring BRCA1/2 mutations (142). Despite profound initial responses, acquired resistance frequently emerges, precipitating disease relapse and therapeutic failure. E3 ubiquitin ligase-mediated regulation of BRCA-associated HR pathways critically contributes to this resistance (143, 144).

**Figure 4:**
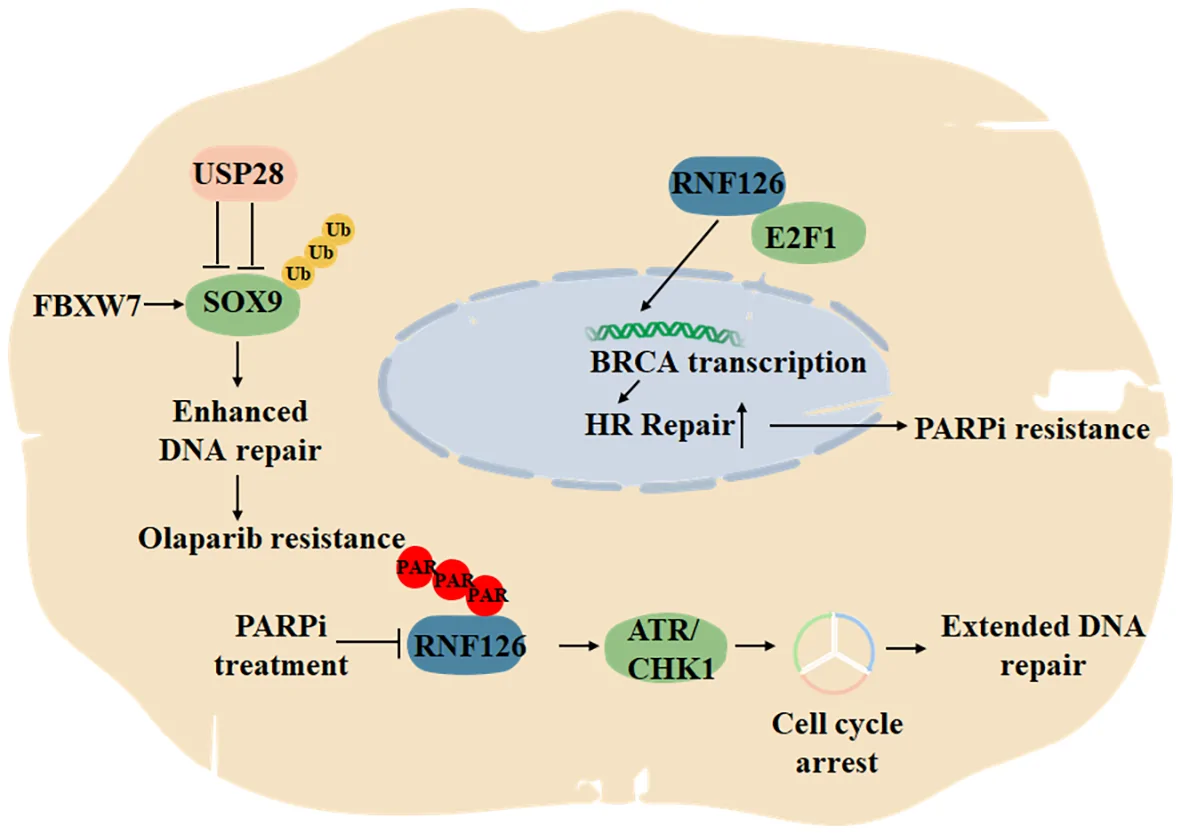
E3 ubiquitin ligases modulate PARP inhibitor sensitivity. The E3 ligase RNF126 interacts with E2F1 to drive BRCA1 transcription and facilitate HR-mediated repair. Upon DNA damage, activated PARP1 generates poly(ADP-ribose) (PAR) chains that PARylate RNF126, targeting it for degradation. Conversely, PARPi treatment suppresses PARP1 activity, preventing RNF126 PARylation and degradation. RNF126 accumulation sustains checkpoint signaling, prolongs G2/M arrest, and extends DNA repair duration, ultimately reducing chemosensitivity. The E3 ligase FBXW7 ubiquitinates SOX9 for degradation and reduce DNA repair, while the deubiquitinating enzyme USP28 stabilizes SOX9 to enhance DNA repair capacity and promote olaparib resistance.

In BRCA-deficient tumors, PARPis induce synthetic lethality by exploiting unrepaired DNA damage. However, resistance can develop through HR restoration via BRCA reversion mutations or epigenetic reprogramming (145, 146). The E3 ubiquitin ligase RNF126 promotes BRCA1 transcription through E2F1 interaction, thereby enhancing HR capacity (147). Upon DNA damage, PARP1-generated poly ADP-ribose (PAR) modifications target RNF126 for degradation, attenuating ATR/CHK1 signaling and enabling G2-M checkpoint bypass. Under PARPi treatment, PARP1 inhibition abrogates RNF126 degradation, causing its accumulation, G2-M arrest prolongation, and extended DNA repair, ultimately conferring resistance (**Figure 4**) (148). While the E3 ligase CHFR conversely sensitizes cells to PARPi by targeting RNF126 for degradation, this highlights the balanced ubiquitination dynamics governing drug response (148). Conversely, RNF126 depletion restores PARPi sensitivity in BRCA-deficient cells, validating E3-mediated repair protein equilibrium as a resistance determinant.

Notably, E3-mediated resistance mechanisms extend beyond BRCA proteins. During olaparib treatment, the E3 ligase FBXW7 mediates the ubiquitination of SOX9 for degradation, while the deubiquitinating enzyme USP28 stabilizes SOX9 to enhance DNA damage repair, promoting olaparib resistance (**Figure 4**) (149). TRIP12-mediated PARP1 ubiquitination and degradation prevents PARPi-induced cytotoxicity, establishing a negative correlation between TRIP12 expression and PARPi sensitivity (150). Combination strategies targeting ubiquitination-regulated resistance are emerging. For instance, USP28 inhibitors (e.g., AZ1) partially reverse PARPi resistance (149). Additionally, ATR inhibitor-PARPi co-administration overcomes HR-driven resistance and represents an active clinical investigation area (151, 152). Thus, integrating multiple E3- and DUB-regulated DNA repair mechanisms with PARPi therapy holds promise for enhancing efficacy and delaying resistance onset.

Collectively, E3 ubiquitin ligases critically govern PARPi resistance through modulation of BRCA-PARP1 protein stability, HR activity, and DNA damage response. Elucidating these ubiquitin system mechanisms illuminates the molecular basis of therapeutic refractoriness and establishes rational targets for combination strategies to optimize PARPi clinical utility in gastrointestinal malignancies.

## 5 E3 ligases modulate drug resistance in gastrointestinal tumors

### 5.1 Gastric cancer

Chemotherapy resistance in gastric cancer (GC) constitutes a formidable clinical challenge that substantially compromises patient outcomes and prognosis. E3 ubiquitin ligases, as critical enzymatic components of the ubiquitin-proteasome system, selectively recognize substrate proteins and orchestrate their ubiquitination-mediated degradation, thereby playing pivotal roles in GC chemo-resistance mechanisms. Recent investigations have elucidated diverse E3 ligase-mediated resistance pathways, principally involving modulation of apoptosis, signal transduction, cell cycle regulation, and ferroptosis (11).

**Figure 5:**
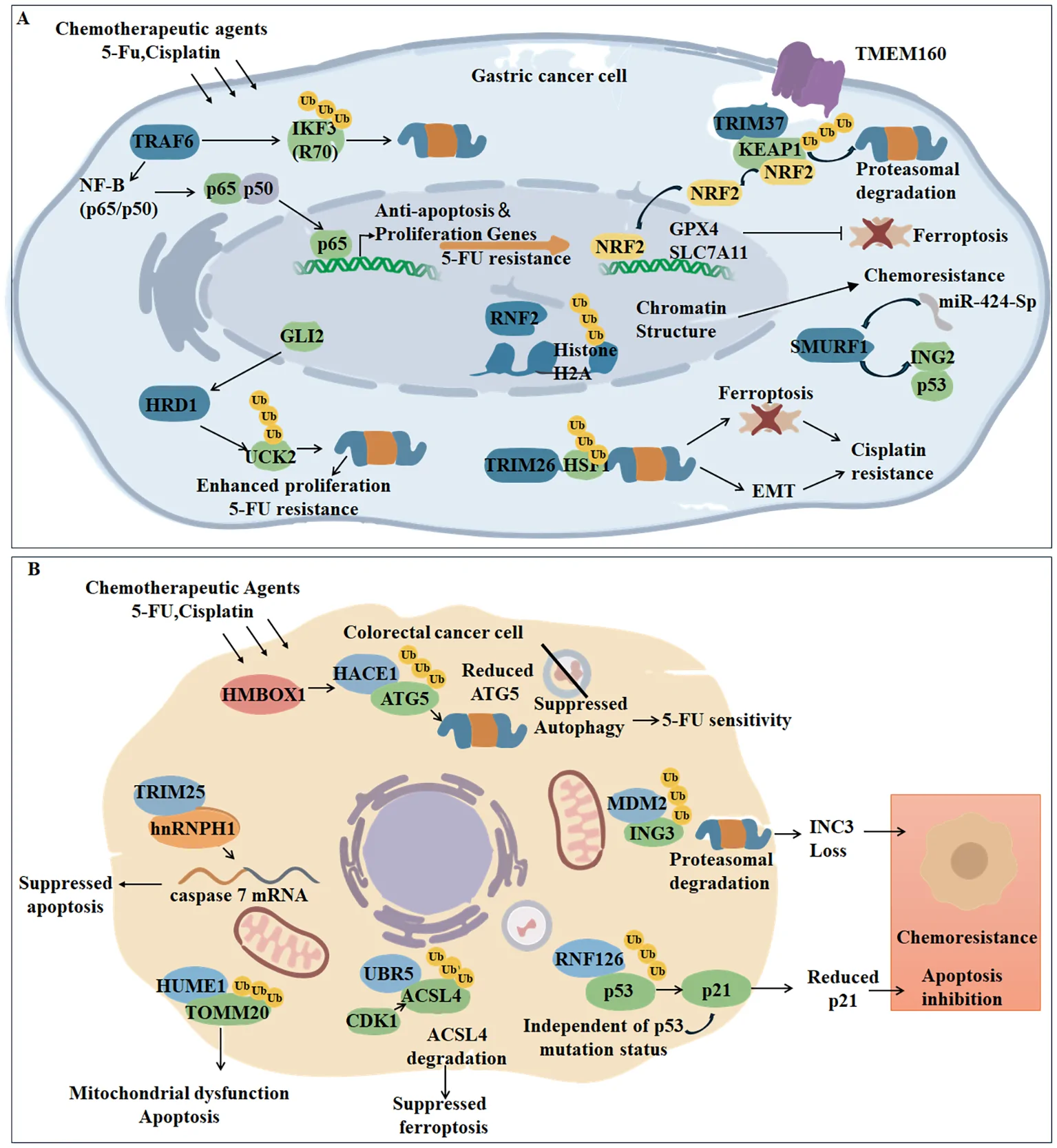
E3 ubiquitin ligases regulate chemotherapeutic response in gastrointestinal malignancies. **(A)** Gastric cancer mechanisms. TRAF6 promotes K48-linked IRF3 ubiquitination at lysine 70, regulating IRF3 stability and facilitating NF-κB p65 nuclear translocation, which activates anti-apoptotic and proliferative gene programs, attenuating 5-FU sensitivity. TMEM160 recruits TRIM37 to mediate K48-linked KEAP1 ubiquitination and degradation, activating NRF2 signaling, which upregulate GPX4 and SLC7A11 expression, thereby suppressing ferroptosis and enhancing chemoresistance. GLI2 upregulates HRD1, which ubiquitinates and degrades UCK2 to promote proliferation and 5-FU resistance. RNF2 mediates ubiquitination of histone H2A, which modulates chromatin states to promote chemo-resistance. TRIM26-mediated HSF1 degradation regulates ferroptosis and epithelial-mesenchymal transition, influencing cisplatin resistance. SMURF1 mediates miR-424-5p-regulated ING2 and p53 degradation, impacting apoptosis and therapeutic response. **(B)** Colorectal cancer mechanisms. HMBOX1 enhances HACE1 transcription, mediating K63-linked ATG5 ubiquitination and degradation, which suppresses autophagy, increasing 5-FU sensitivity. MDM2 promotes ING3 degradation, facilitating proliferation and apoptotic evasion to confer chemoresistance. RNF126 induces p53 ubiquitination and degradation, downregulating p21 to inhibit apotosis, conferring drug resistance. CDK1-phosphorylated ACSL4 recruits UBR5 for ubiquitination and degradation, suppressing ferroptosis and promoting oxaliplatin resistance. HUWE1 ubiquitinates TOMM20, impairing mitochondrial function, elevating ROS, and inducing apoptosis to sensitize cells to oxaliplatin.

First, TRAF6 promotes K48-linked ubiquitination of interferon regulatory factor 3 (IRF3) at lysine 70, thereby modulating IRF3 stability. This facilitates NF-κB-p65 nuclear translocation, activates anti-apoptotic and proliferative gene programs, and attenuates GC cell sensitivity to 5-fluorouracil (5-FU) (153). TRAF6 inhibition significantly suppresses drug-resistant cell proliferation and tumor growth, establishing the TRAF6-IRF3 axis as a critical mediator of GC chemo-resistance (**Figure 5A**) (154).

Second, TMEM160 recruits the E3 ligase TRIM37 to promote K48-linked ubiquitination and proteasomal degradation of KEAP1. This activates NRF2 signaling, upregulating glutathione peroxidase 4 (GPX4) and cystine transporter SLC7A11 expression, thereby suppressing ferroptosis and enhancing chemo-resistance (**Figure 5A**) (155, 156). Combined TMEM160 targeting with chemotherapy synergistically inhibits GC cell growth *in vitro* and *in vivo*, identifying the TRIM37-KEAP1-NRF2 axis as a promising therapeutic target.

Third, the transcription factor GLI2 upregulates HRD1 expression, promoting ubiquitin-mediated UCK2 degradation. This enhances GC cell proliferation and 5-FU resistance, whereas GLI2 knockdown restores 5-FU sensitivity (**Figure 5A**) (157).

Additional E3 ligases also contribute to GC chemo-resistance through distinct mechanisms. RNF2, as a histone H2A E3 ligase, modulates chromatin states to promote resistance (158); TRIM26 targets heat shock factor 1 (HSF1) for ubiquitination and degradation, thereby regulating ferroptosis and epithelial-mesenchymal transition (EMT) to influence cisplatin sensitivity (159); SMURF1 mediates cisplatin resistance through miR-424-5p-regulated targeting of ING2 and p53 (**Figure 5A**) (160).

Collectively, E3 ubiquitin ligases orchestrate GC chemo-resistance through multifaceted mechanisms encompassing transcription factor regulation, signaling pathway modulation, apoptotic evasion, and ferroptosis suppression. Comprehensive characterization of E3-mediated ubiquitination networks will facilitate the development of precision therapeutics to reverse resistance, thereby improving clinical outcomes and survival for GC patients.

### 5.2 Colorectal cancer

Colorectal cancer (CRC), a prevalent gastrointestinal malignancy, remains primarily dependent on systemic chemotherapy for patients with unresectable or advanced disease. Acquired chemo-resistance, however, substantially limits therapeutic efficacy and represents a critical determinant of patient prognosis (161). E3 ubiquitin ligases, as central regulators of protein homeostasis and signal transduction, have emerged as pivotal mediators of CRC chemo-resistance.

First, HMBOX1 exemplifies transcriptional regulation of E3-mediated resistance mechanisms. Downregulated in drug-resistant cells, HMBOX1 correlates with adverse clinical outcomes. Mechanistically, HMBOX1 drives HACE1 transcription, which mediates K63-linked ubiquitination–dependent regulation of autophagy-related protein ATG5. ATG5 suppression inhibits autophagic flux, thereby attenuating autophagy-mediated 5-FU resistance (162).

Second, MDM2 targets the transcription factor ING3 for ubiquitin-proteasome degradation, fostering CRC cell proliferation and apoptotic evasion, conferring chemo-resistance (163). Additionally, the E3 ligase RNF126, highly expressed in CRC, promotes p53 ubiquitination and degradation, thereby reducing p21 expression and enhancing proliferation, metastatic potential, and drug resistance. Notably, RNF126-p53 complex formation and subsequent p53 degradation occur independently of p53 mutation status, underscoring RNF126’s critical role in governing wild-type p53 stability. Clinically, RNF126 overexpression correlates with tumor burden, lymph node metastasis, and advanced pathological stage (**Figure 5B**) (164).

Furthermore, platinum-based resistance also involves ferroptosis modulation. CDK1-mediated phosphorylation of fatty acid synthase ACSL4 recruits UBR5 to mediate ACSL4 ubiquitination and degradation, thereby suppressing ferroptosis and promoting oxaliplatin resistance (**Figure 5B**). Conversely, CDK1 inhibition restores ACSL4 levels, reactivates ferroptosis, and enhances chemosensitivity (165). HUWE1, another E3 ligase, conversely sensitizes CRC cells to oxaliplatin by directly ubiquitinating mitochondrial outer membrane protein TOMM20, which impairs mitochondrial integrity, elevates reactive oxygen species (ROS) production, and triggers apoptosis (**Figure 5B**) (166). Additionally, by binding heterogeneous nuclear ribonucleoprotein H1 (hnRNPH1), TRIM25 destabilizes caspase-7 mRNA, thereby reducing caspase-7 expression, suppressing apoptosis, and enhancing chemo-resistance (**Figure 5B**). This TRIM25-hnRNPH1-caspase-7 axis reveals an anti-apoptotic mechanism, suggesting TRIM25 targeting as a strategy to overcome resistance (167).

Collectively, E3 ubiquitin ligases orchestrate complex chemo-resistance networks in CRC through ubiquitin-mediated degradation of key regulatory proteins, thereby modulating autophagy, apoptosis, ferroptosis, transcriptional programs, and signal transduction. Therapeutic strategies targeting specific E3 ligases and their downstream substrates hold substantial promise for overcoming chemo-resistance and improving clinical outcomes in CRC.

## 6 Overcoming tumor resistance with E3 ligase inhibitors

### 6.1 Clinical applications of MDM2 inhibitors

As delineated above, MDM2 functions as the principal negative regulator of p53, promoting p53 ubiquitination and proteasomal degradation through direct physical interaction, thereby attenuating p53-mediated tumor suppression. This MDM2-p53 axis represents a critical driver of tumorigenesis and chemoresistance across diverse malignancies (168). Nutlin-3, a prototype small-molecule inhibitor, occupies the p53-binding pocket of MDM2, competitively disrupting MDM2-p53 association and restoring p53 transcriptional activity to induce cell cycle arrest and apoptosis (88). Additionally, Nutlin-3 activates p53-independent cell death pathways, further enhancing antitumor efficacy (169). Clinical translation, however, remains constrained by acquired resistance, dose-limiting toxicities, and hematologic adverse events (168, 170).

Structural derivatives including RG7112, idasanutlin (RG7388), and AMG-232 (KRT-232) have advanced through various phases of clinical development across multiple tumor types, including gastrointestinal malignancies (170, 171). These agents demonstrate activity in wild-type p53 tumors; however, monotherapy frequently yields suboptimal durable responses, necessitating combination strategies (172).

Nutlin-3-MEK inhibitor combinations exhibit synergistic activation of p53 and enhanced antitumor activity in KRAS-mutant colorectal and gastric cancer cells (173). Concurrently, Nutlin-3-immune checkpoint inhibitor combinations are under active investigation to circumvent resistance and amplify therapeutic efficacy. Notably, Nutlin-3 exhibits reduced propensity for secondary resistance compared to conventional cytotoxic agents, enhancing chemosensitivity through p53 pathway activation while concomitantly suppressing ABC transporter-mediated drug efflux, manifesting resistance reversal phenotypes in certain cellular contexts (174, 175). Furthermore, MDM2 inhibitors modulate tumor microenvironment composition and immune cell function, providing a mechanistic rationale for combination immunotherapy (175).

Collectively, Nutlin-3 and its derivatives substantiate the translational potential of E3 ligase inhibition for reversing clinical chemo-resistance through MDM2-p53 axis disruption and p53 restoration. Despite challenges including resistance emergence and hematologic toxicity, ongoing clinical trials indicate broad applicability across malignancies, with promise in rational combination regimens. Integrating precision medicine strategies with next-generation inhibitor design, MDM2-targeted therapeutics hold substantial promise as a transformative strategy to overcome chemotherapy resistance in gastrointestinal tumors and beyond.

### 6.2 Cullin-RING E3 ligase inhibitors

Cullin-RING ubiquitin ligases (CRLs) constitute the largest E3 ligase family, orchestrating approximately 20% of intracellular proteolytic events. These complexes govern diverse biological processes encompassing cell cycle progression, signal transduction, and oncogenesis (176, 177). CRL activation requires NEDD8-mediated neddylation of Cullin scaffolds, rendering this post-translational modification a prime target for therapeutic intervention.

MLN4924 (pevonedistat), the prototypic NEDD8-activating enzyme (NAE) inhibitor, blocks Cullin neddylation to suppress CRL activity, demonstrating promising antitumor efficacy in preclinical and early clinical investigations (178, 179). Additionally, the natural product gossypol directly binds Cullin1 and Cullin5 complexes to inhibit neddylation, causing accumulation of CRL1 and CRL5 substrates and enhanced cytotoxicity (176, 180). Concurrently, small-molecule inhibitors targeting the DCN1-UBE2M protein-protein interaction selectively suppress neddylation of specific Cullin isoforms, exhibiting excellent selectivity and antitumor activity (181–184).

High-throughput screening has identified 33–11 and KH-4–43 as direct CRL4 core complex binders. These agents inhibit CRL4-mediated substrate ubiquitination, leading to CDT1 accumulation, replication stress-induced apoptosis, and significant antitumor effects (177, 185). These findings expand the CRL inhibitor molecular toolkit and establish rationale for developing CRL4-specific therapeutics.

Suramin and SMER3 represent pioneering CRL inhibitors. Suramin broadly suppresses ubiquitin ligase family members by blocking substrate recognition or ubiquitin transfer (186). SMER3 specifically targets CRL complex components to inhibit ubiquitin conjugation, compromising tumor cell viability and proliferation (187). Although their detailed molecular mechanisms require further elucidation, both agents demonstrate clinical translational potential.

Collectively, CRL inhibitors restore substrate protein stability and suppress malignant proliferation through multiple mechanisms: Neddylation blockade, E2-E3 interaction disruption, and direct core complex engagement. These diverse strategies hold substantial promise for overcoming CRL-driven oncogenic programs.

### 6.3 Other E3 ubiquitin ligase inhibitors

Beyond established CRL and HECT-targeting agents, novel inhibitors such as Pro-Tame and Apcin selectively target the anaphase-promoting complex/cyclosome (APC/C), a RING-type E3 ligase critical for cell cycle regulation (80, 188). Pro-Tame, a small-molecule RING domain competitor, mimics endogenous inhibitors to suppress APC/C ubiquitin ligase activity, thereby blocking substrate ubiquitination and inducing mitotic arrest and apoptotic cell death (188). Apcin operates through a distinct mechanism, competitively binding APC/C substrates to prevent their recognition and degradation, thereby modulating cell cycle progression (189). Synergistic application of both agents significantly attenuates cancer cell proliferation in preclinical models, particularly in cells refractory to conventional chemotherapy (177).

APC/C hyperactivation is intimately linked to chemo-resistance in gastrointestinal malignancies, positioning Pro-Tame and Apcin as promising modalities to circumvent therapeutic refractoriness. Additionally, CRL4 inhibitors (33-11, KH-4-43) demonstrate potential to induce apoptosis through CDT1 stabilization, expanding the E3 inhibitor developmental pipeline (177, 190).

Despite promising preclinical activity, these agents require optimization of safety profiles, target selectivity, and pharmacokinetic properties prior to clinical translation. Future priorities include: (i) enhancing inhibitor affinity and isoform selectivity to minimize off-target effects; (ii) developing rational combination regimens with existing chemotherapeutics to amplify efficacy and overcome resistance; and (iii) leveraging structural biology and computational drug design to target E3 catalytic cores with improved precision. These strategies collectively advance the clinical utility of E3-directed therapeutics in gastrointestinal oncology.

Overall, emerging E3 inhibitors, including APC/C-targeting Pro-Tame and Apcin, offer innovative approaches to dissect and target dysregulated protein degradation in gastrointestinal tumors. By precisely modulating critical ubiquitin-dependent pathways, these compounds hold substantial promise for overcoming chemoresistance and improving patient outcomes.

### 6.4 Clinical translation of UPS-targeted therapies

The clinical relevance of targeting the ubiquitin–proteasome system (UPS) has been demonstrated by proteasome inhibitors. Bortezomib, a clinically approved proteasome inhibitor, is indicated for multiple myeloma and mantle cell lymphoma, establishing the UPS as a druggable therapeutic axis (191, 192). More selective UPS-targeted strategies have also entered clinical development. MDM2 inhibitors, such as idasanutlin, have shown promising antitumor activity in preclinical studies, including *in vitro* experiments and mouse xenograft models, particularly in tumors retaining wild-type TP53 (22, 171). However, clinical translation has proven challenging.

In the phase III MIRROS trial in patients with relapsed or refractory acute myeloid leukemia (AML), the combination of idasanutlin and cytarabine improved response rates but failed to significantly improve overall survival compared with cytarabine alone (22). These findings highlight the translational limitations of MDM2-targeted therapies and underscore the importance of biomarker-guided patient selection, particularly with respect to TP53 status. Future development of E3 ligase-targeted therapies in gastrointestinal malignancies will likely require rational combination strategies and molecular stratification approaches to maximize therapeutic benefit (22).

To provide a concise overview of the current clinical landscape of E3 ligase-targeting therapeutics, representative agents and their translational progress are summarized in **Table 2**. Although several E3-directed strategies have entered clinical development, therapeutic resistance remains a major challenge. For MDM2 inhibitors, resistance frequently arises through TP53 mutations, MDMX overexpression, or defects in downstream p53 signaling, thereby limiting the efficacy of p53 reactivation strategies (88, 168). Similarly, resistance to cereblon (CRBN)-targeting molecular glues may result from CRBN downregulation or mutation, leading to impaired neo-substrate degradation (22, 193). Emerging evidence also indicates that PROTAC-based therapeutics can be compromised by alterations in E3 ligase expression, target mutations, or disruption of productive ternary complex formation (193). Consequently, biomarker-guided patient selection, rational combination therapies involving DDR inhibitors or immunotherapy, and the development of next-generation degraders are being actively explored to enhance therapeutic efficacy and overcome acquired resistance (22, 193).

**Table 2:** Representative E3 ligase-targeting therapeutics and resistance mechanisms.

Drug	Clinical status	Trial number	Targeted E3 ligase	Resistance mechanism	Resistance-overcoming strategy
Idasanutlin (RG7388)	Phase III completed	NCT02545283	MDM2	TP53 mutation, MDMX overexpression, defective p53 signaling	Patient selection based on TP53 WT status; combination with chemotherapy or immunotherapy
AMG-232 (KRT-232)	Phase II	NCT03725436	MDM2	TP53 loss and compensatory survival signaling	Combination with DDR-targeted therapies or immune checkpoint blockade
RG7112	Phase I/II	NCT01605526	MDM2	Acquired p53 pathway defects	Combination strategies with chemotherapy
Pevonedistat (MLN4924)	Phase III	NCT03268954	Cullin-RING E3 ligases (via NAE inhibition)	Reactivation of alternative ubiquitin pathways and adaptive DDR signaling	Combination with chemotherapy, PARP inhibitors, or ATR inhibitors
Lenalidomide	Approved	Multiple trials	CRBN (CRL4CRBN)	CRBN loss/downregulation and impaired neo-substrate degradation	Development of next-generation CRBN modulators
Pomalidomide	Approved	Multiple trials	CRBN (CRL4CRBN)	CRBN mutation or reduced substrate recruitment	Combination therapy and next-generation molecular glues
ARV-110 (Bavdegalutamide)	Phase I/II	NCT03888612	CRBN/VHL recruiting PROTAC	E3 ligase downregulation, target mutation, impaired ternary complex formation	Alternative E3 recruiters and next-generation PROTAC design

### 6.5 PROTACs and molecular glue degraders proteolysis-targeting chimeras and molecular glue degraders represent major advances in therapeutic strategies that exploit the ubiquitin–proteasome system

PROTACs are heterobifunctional molecules that simultaneously bind a protein of interest and an E3 ubiquitin ligase, thereby inducing proximity-dependent ubiquitination and proteasomal degradation of disease-driving proteins (194, 195). Unlike conventional inhibitors, PROTACs eliminate the target protein itself and may therefore overcome resistance caused by persistent scaffolding functions or incomplete enzymatic inhibition (194, 196). ARV-110 (bavdegalutamide) is a representative clinically investigated PROTAC that targets the androgen receptor by recruiting an E3 ligase complex and promoting receptor degradation (194). As one of the first PROTAC degraders to enter clinical evaluation, it has been investigated in metastatic castration-resistant prostate cancer and provides proof-of-concept that E3 ligase-recruiting degradation strategies can be translated into clinical settings (194, 197).

Molecular glue degraders act through a related but mechanistically distinct strategy. Instead of using a bifunctional linker, molecular glues enhance or create interactions between an E3 ligase and a neo-substrate (198). The immunomodulatory drugs lenalidomide and pomalidomide are classical examples that bind cereblon (CRBN), a substrate receptor of the CRL4 E3 ligase complex, and promote degradation of neo-substrates such as IKZF1 and IKZF3 (24, 193). These agents demonstrate that pharmacological rewiring of E3 ligase substrate specificity can produce clinically meaningful antitumor effects (24, 193).

For gastrointestinal malignancies, PROTACs and molecular glues may provide new opportunities to target proteins that are difficult to inhibit using traditional small molecules, including transcription factors, scaffold proteins, and context-dependent resistance drivers (194, 196). However, their clinical application will require careful optimization of E3 ligase selection, tissue specificity, degradation selectivity, pharmacokinetics, toxicity, and biomarker-guided patient stratification (25, 195). Moreover, emerging degradation-based modalities face additional development challenges, including limited oral bioavailability, suboptimal pharmacokinetic profiles, tissue-specific E3 ligase dependency, and acquired resistance mechanisms. Addressing these limitations will be essential for successful clinical translation (25, 195).

**Figure 6:**
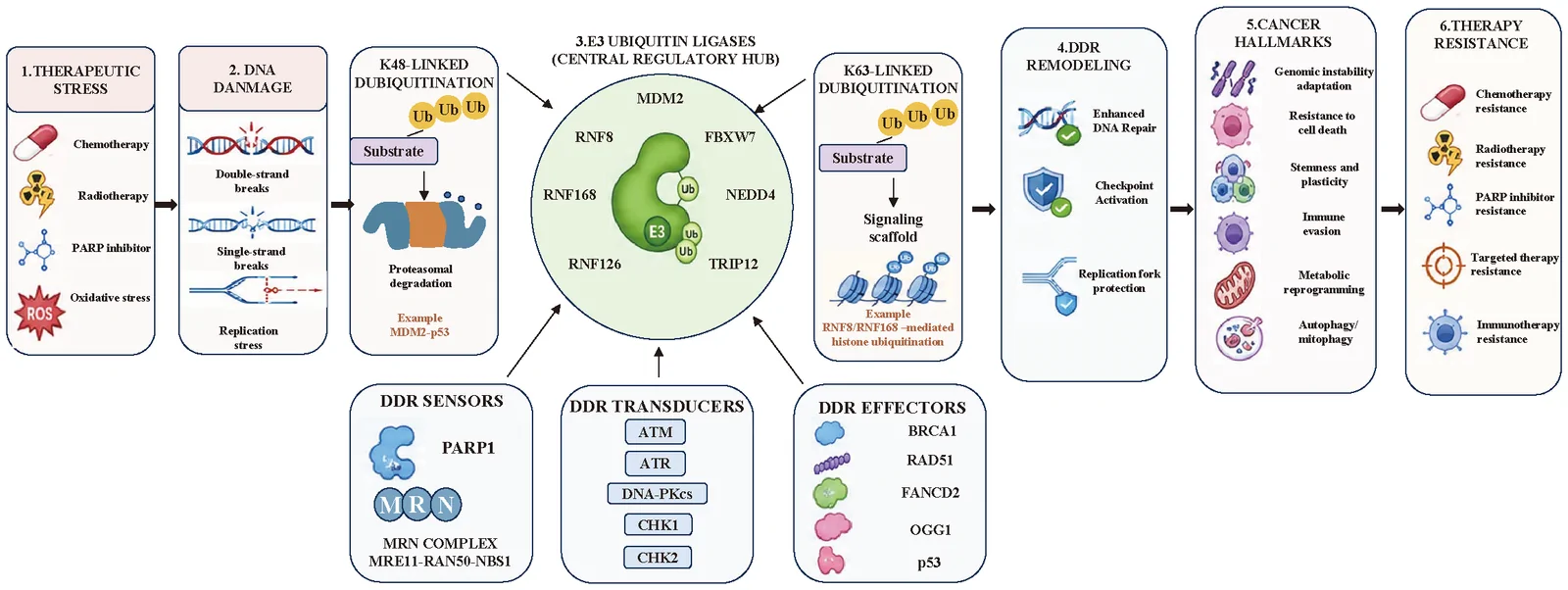
E3 ubiquitin ligase-mediated mechanisms of therapeutic resistance in gastrointestinal tumors.​ E3 ubiquitin ligases function as central regulators of therapeutic resistance by modulating DNA damage response (DDR) signaling, oncogenic pathways, and immune regulatory networks in gastrointestinal malignancies. In chemotherapy resistance, E3 ligases such as MDM2, FBXW7, and NEDD4 promote tumor cell survival through p53 degradation, activation of oncogenic signaling, and PI3K–AKT pathway stimulation, thereby contributing to resistance to cisplatin, 5-fluorouracil (5-FU), and oxaliplatin. In radiotherapy resistance, RNF8 and RNF168 facilitate DNA double-strand break (DSB) repair and ATM-dependent DDR signaling, enhancing cellular tolerance to radiation-induced DNA damage. In PARP inhibitor (PARPi) resistance, RNF126 and TRIP12 promote homologous recombination (HR) restoration through the regulation of BRCA1 and RAD51, thereby reducing PARPi sensitivity. In targeted therapy resistance, dysregulation of FBXW7 sustains Notch, Wnt/β-catenin, and mTOR signaling pathways, promoting therapeutic escape. In immunotherapy resistance, E3 ligases involved in immune checkpoint regulation, including β-TrCP, SPOP, and members of the Cullin family, modulate PD-L1 stability and immune signaling, facilitating immune evasion and resistance to immune checkpoint inhibitors (ICIs). Collectively, these ubiquitin-dependent mechanisms enhance DNA repair capacity, activate pro-survival signaling pathways, promote metabolic and autophagic adaptation, and suppress antitumor immunity, ultimately leading to treatment failure, tumor recurrence, disease progression, and poor clinical outcomes.

To provide a comprehensive overview of the resistance mechanisms discussed throughout this review, **Figure 6** summarizes the major pathways through which E3 ubiquitin ligases contribute to therapeutic resistance in gastrointestinal tumors. As illustrated in **Figure 6**, aberrant E3 ligase activity influences multiple biological processes involved in treatment response, including DNA damage repair, checkpoint regulation, apoptosis, cancer stemness, metabolic reprogramming, autophagy, and immune modulation. These mechanisms do not function independently but instead form an interconnected regulatory network that promotes tumor cell survival and therapeutic adaptation under treatment-induced stress. Consequently, E3 ubiquitin ligases represent critical molecular hubs linking DDR remodeling to diverse resistance phenotypes and may serve as promising therapeutic targets for overcoming treatment resistance in gastrointestinal malignancies.

### 6.6 Challenges and future directions for clinical translation of E3-targeted therapies

Despite substantial advances in understanding the biological functions of E3 ubiquitin ligases, several challenges remain before E3-targeted therapies can be broadly implemented in clinical practice. One major challenge is the identification of reliable biomarkers for patient stratification. Because the therapeutic effects of E3 ligase inhibitors are highly context-dependent, molecular characteristics such as TP53 status, DDR proficiency, E3 ligase expression levels, and tumor subtype may critically influence treatment responses (22, 171). For example, the efficacy of MDM2 inhibitors largely depends on the presence of functional wild-type p53, highlighting the importance of biomarker-guided patient selection (171, 194).

Another important consideration is the development of rational combination strategies. Given the central role of E3 ligases in regulating DNA repair, apoptosis, immune signaling, stemness, and metabolic adaptation, monotherapy may not be sufficient to achieve durable clinical responses. Increasing evidence suggests that combining E3-targeted agents with chemotherapy, PARP inhibitors, radiotherapy, targeted therapies, or immunotherapy may enhance therapeutic efficacy by simultaneously disrupting multiple resistance mechanisms (22, 194, 199). Such combination approaches may be particularly valuable in gastrointestinal malignancies characterized by complex and heterogeneous resistance networks.

In addition, acquired resistance to E3-targeted therapies is emerging as a significant challenge. Similar to other targeted therapies, tumor cells can develop resistance through genetic and non-genetic mechanisms. For example, mutations or downregulation of cereblon (CRBN) have been associated with resistance to immunomodulatory drugs (200), while alterations in substrate recognition, compensatory activation of parallel signaling pathways, or rewiring of ubiquitin-dependent regulatory networks may also limit treatment efficacy (194, 195). Understanding these resistance mechanisms will be essential for the design of next-generation E3 ligase-targeted therapeutics.

Collectively, future clinical development of E3-targeted therapies should integrate biomarker-guided patient stratification, rational combination strategies, and resistance-monitoring approaches. Such efforts will facilitate precision targeting of the ubiquitin system and accelerate translation of mechanistic discoveries into effective therapeutic interventions for gastrointestinal malignancies (22). The mechanistic and therapeutic evidence supporting E3-targeted interventions is derived from *in vitro*, *in vivo*, and early clinical studies. While preclinical models support efficacy and mechanism, limitations such as variable substrate detection, transient ubiquitination events, and reproducibility issues necessitate cautious interpretation (195).

## 7 Context-dependent functions and unresolved controversies of E3 ligases

Despite substantial evidence supporting the role of E3 ubiquitin ligases in therapy resistance, emerging studies indicate that their biological functions are highly context-dependent and cannot always be generalized across tumor types, genetic backgrounds, or therapeutic settings (22, 66, 201). Consequently, several E3 ligases exhibit apparently contradictory effects on treatment responses, highlighting the complexity of ubiquitin-mediated regulation within the DNA damage response (DDR) network.

MDM2 represents a classic example of context-dependent E3 ligase activity. In tumors harboring wild-type TP53, MDM2-mediated ubiquitination promotes p53 degradation, thereby suppressing apoptosis and facilitating resistance to DNA-damaging therapies (23, 171). Accordingly, pharmacological inhibition of the MDM2–p53 interaction has demonstrated therapeutic benefit in multiple preclinical models (171). However, in TP53-mutant tumors, the biological consequences of MDM2 inhibition are often attenuated or even absent because p53-dependent apoptotic signaling is already compromised (171). Furthermore, MDM2 regulates non-p53 substrates involved in DNA repair, cell-cycle progression, and immune signaling, suggesting that its overall contribution to therapy resistance may vary according to molecular context (16, 23). These observations indicate that TP53 status is likely a critical determinant of therapeutic responses to MDM2-targeted strategies.

Similar complexity has been reported for FBXW7. While FBXW7 is generally considered a tumor suppressor through degradation of oncogenic substrates such as c-Myc, Cyclin E, and Notch, recent studies suggest that FBXW7 loss may have distinct consequences depending on treatment modality and tumor lineage (202). In some settings, FBXW7 deficiency promotes genomic instability and therapeutic resistance, whereas in others it may increase replication stress and sensitize cancer cells to DNA-damaging agents (202, 203). Such findings imply that the relationship between FBXW7 activity and drug response is not universally linear.

TRIP12 provides another example of context-dependent DDR regulation. Although TRIP12-mediated control of DNA repair proteins has been associated with enhanced resistance to genotoxic therapies, several studies indicate that excessive suppression of TRIP12 may induce genomic instability beyond a tolerable threshold, thereby impairing tumor cell fitness (204). Consequently, the therapeutic window for targeting TRIP12 remains incompletely defined.

RNF43 provides a clear example of context-dependent activity in gastrointestinal cancers. While RNF43 is generally considered a tumor suppressor through negative regulation of Wnt/β-catenin signaling in colorectal cancers, recent studies indicate that in other contexts, such as pancreatic or gastric tumors with distinct mutational backgrounds, RNF43 loss or mutation can paradoxically enhance Wnt-independent survival pathways, contributing to therapy resistance (57). These observations highlight that the functional consequences of RNF43 alterations are highly context-dependent, influenced by tumor lineage, co-occurring mutations, and microenvironmental cues (205).

Collectively, these observations suggest that E3 ubiquitin ligases should not be classified simply as resistance-promoting or resistance-suppressing factors. Instead, their biological effects are determined by a combination of factors, including tumor lineage, genomic landscape, DDR status, treatment modality, and tumor microenvironment (22, 66). Future investigations should therefore focus on defining context-specific E3 signaling networks rather than evaluating individual ligases in isolation. Such an approach may improve patient stratification and facilitate the development of precision therapeutic strategies targeting the ubiquitin system.

Another important consideration is the substantial heterogeneity that exists among different gastrointestinal malignancies. Although GI cancers are frequently discussed as a collective disease group, accumulating evidence indicates that the biological functions and clinical significance of individual E3 ubiquitin ligases are often tumor-type specific (22, 202, 205). FBXW7 provides a representative example of this heterogeneity. FBXW7 mutations occur at relatively high frequencies in colorectal cancer and are associated with aberrant activation of Notch, c-Myc, and Cyclin E signaling pathways, contributing to tumor progression, stemness, and therapeutic resistance (203). In contrast, the prevalence, functional consequences, and therapeutic implications of FBXW7 alterations appear to differ substantially in gastric and esophageal cancers (203, 206). Similar tumor-specific differences have been reported for multiple E3 ligases involved in DDR regulation, immune signaling, metabolic adaptation, and Wnt/β-catenin signaling networks in colorectal cancer (22, 202, 207). These observations suggest that E3 ligase function is strongly influenced by tissue lineage, mutational landscape, epigenetic context, and microenvironmental conditions (22, 206). Consequently, therapeutic strategies targeting E3 ligases will likely require tumor-specific biomarker-guided approaches rather than uniform application across all gastrointestinal malignancies (22, 202).

## 8 Future perspectives

### 8.1 AI-driven drug discovery

Artificial intelligence (AI) and machine learning technologies are emerging as powerful tools for accelerating the discovery of UPS-targeted therapeutics (194, 208). AI-based approaches can facilitate prediction of E3 ligase–substrate interactions, identification of novel degron motifs, optimization of PROTAC linker design, and virtual screening of molecular glue degraders (197, 208). Integration of multi-omics datasets with structural biology and deep learning algorithms may further improve target prioritization and enable more precise therapeutic development (194, 209).

### 8.2 Precision oncology

Future clinical implementation of E3-targeted therapies will likely depend on biomarker-guided patient stratification (22). Integration of TP53 status, DDR proficiency, E3 ligase expression patterns, and tumor-specific molecular features may improve patient selection and therapeutic efficacy (22, 142, 171).

### 8.3 Next-generation degradation technologies

Beyond conventional E3 ligase inhibitors, emerging technologies including PROTACs, molecular glue degraders, lysosome-targeting chimeras (LYTACs), autophagy-targeting chimeras (AUTACs), and RNA-targeting degradation platforms may substantially expand the therapeutic landscape of the ubiquitin system (194, 198, 210, 211).

## 9 Conclusion and outlook

In recent years, the pivotal role of E3 ubiquitin ligases in GI cancer chemoresistance has become increasingly recognized, making them a central focus in cancer biology and therapeutic development. As core components of the ubiquitin–proteasome system, E3 ligases selectively direct target protein degradation, thereby modulating proliferation, apoptosis, DNA repair, and signal transduction, ultimately governing chemotherapeutic sensitivity. Extensive investigations demonstrate that E3 ligase expression and function exhibit substantial heterogeneity across gastric, colorectal, and esophageal malignancies, with distinct family members intimately associated with resistance phenotypes. These insights not only advance mechanistic understanding but also establish a foundation for innovative therapeutic strategies. This review integrates E3 ligases, DDR remodeling, and hallmark-associated resistance phenotypes in gastrointestinal tumors, providing a framework that elucidates molecular mechanisms and supports the rational development of E3-targeted therapeutic strategies.

As core components of the ubiquitin–proteasome system (UPS), E3 ligases selectively direct target protein degradation, thereby modulating proliferation, apoptosis, DNA repair, and signal transduction, ultimately governing chemotherapeutic sensitivity. Extensive investigations demonstrate that E3 ligase expression and function exhibit substantial heterogeneity across gastric, colorectal, and esophageal malignancies, with distinct family members closely associated with resistance phenotypes. These insights not only advance mechanistic understanding but also establish a foundation for innovative therapeutic strategies. This review integrates E3 ligases, DDR remodeling, and hallmark-associated resistance phenotypes in gastrointestinal tumors, providing a framework that elucidates molecular mechanisms and supports the rational development of E3-targeted therapeutic strategies.

Notably, accumulating evidence indicates that E3 ubiquitin ligases should not be viewed simply as universally resistance-promoting or resistance-suppressing factors. The biological consequences of E3 ligase activity are highly context-dependent and may vary according to tumor lineage, TP53 status, DDR proficiency, treatment modality, and microenvironmental conditions. For example, the therapeutic impact of MDM2 inhibition differs substantially between TP53-wild-type and TP53-mutant tumors, highlighting the importance of molecular context in determining treatment responses. Similarly, RNF43 exhibits dual roles in Wnt signaling depending on tumor context, emphasizing the need for careful mechanistic interpretation and context-specific therapeutic targeting of E3 ligases. Future studies should therefore move beyond single-substrate analyses and focus on defining context-specific E3 regulatory networks that support biomarker-guided patient stratification and precision therapeutic interventions.

E3 ligase-targeted therapeutics hold significant translational potential. First, the inherent substrate specificity of E3 ligases renders them attractive therapeutic targets, enabling precise modulation of resistance-associated proteins while minimizing off-target effects. Second, E3 inhibitors or modulators can reverse resistance phenotypes, thereby potentiating the efficacy of conventional chemotherapy. However, clinical translation faces substantial challenges: current small-molecule inhibitors, despite promising preclinical activity, require optimization of selectivity, metabolic stability, and safety profiles. Additionally, the vast diversity and functional complexity of the E3 ligase superfamily necessitate deeper mechanistic investigation to precisely identify targetable nodes and downstream effectors. A key concept emerging from current evidence is that E3 ubiquitin ligases function as central coordinators linking ubiquitination-dependent proteostasis, DDR network remodeling, and hallmark-associated therapeutic adaptation. By regulating DNA damage sensing, checkpoint activation, repair pathway selection, and cell-fate determination, E3 ligases reshape the cellular response to therapeutic stress. This systems-level perspective explains why mechanistically distinct E3 ligases may converge on similar resistance phenotypes, including enhanced DNA repair, apoptosis suppression, checkpoint adaptation, stemness maintenance, and immune evasion.

Taken together, available evidence suggests that E3 ligases exhibit context-dependent functions across GI cancer subtypes and patient populations. E3 ligases that promote tumorigenesis and therapeutic resistance in gastric cancer may exhibit distinct biological functions and regulatory mechanisms in colorectal cancer. This heterogeneity underscores the need for multi-omics approaches and precision medicine frameworks to dissect E3 ligase regulatory networks. Furthermore, current research has predominantly focused on the effects of individual E3 ligases. Future studies should place greater emphasis on E3 ligase interactions within broader molecular networks and explore rational combination strategies. In particular, synergistic regimens integrating chemotherapy, targeted therapies, and immunotherapy may maximize therapeutic benefit.

Advancing the clinical development of E3 ligase-targeted therapies represents an important priority for improving outcomes in GI cancers. Key strategies include: (i) expediting the discovery and optimization of highly selective E3 inhibitors with favorable pharmacokinetic and pharmacodynamic properties; (ii) conducting multicenter, adequately powered preclinical and clinical studies to systematically evaluate the efficacy and safety of both monotherapy and combination regimens; and (iii) identifying biomarkers based on E3 ligase expression profiles and functional states to facilitate personalized therapeutic selection and improve understanding of ubiquitin-dependent regulatory networks.

From a translational perspective, clinical experience with UPS-targeted therapies further supports the therapeutic potential of this system. Proteasome inhibitors such as bortezomib have already demonstrated that pharmacological disruption of ubiquitin-dependent protein turnover can produce clinically meaningful antitumor effects. Meanwhile, E3 ligase-directed strategies, including MDM2 antagonists such as idasanutlin, have entered clinical evaluation, although trial outcomes also highlight the challenges of patient selection, toxicity management, and context-dependent treatment responses. Therefore, future development of E3-targeted therapies in gastrointestinal malignancies should incorporate biomarker-guided patient stratification based on TP53 status, DDR proficiency, and tumor-specific molecular characteristics, together with rational combination approaches involving chemotherapy, DDR inhibitors, immunotherapy, or metabolic interventions. While extensive preclinical data support the mechanistic roles of E3 ligases in gastrointestinal cancers, translation of these findings into clinical benefit remains limited, emphasizing the need for careful evaluation of evidence from *in vitro*, *in vivo*, and clinical studies, as well as attention to reproducibility and experimental limitations.

Collectively, the central role of E3 ligases in GI cancer chemoresistance and their therapeutic potential are now widely recognized. Future efforts integrating mechanistic insights, inhibitor optimization, and rational combination strategies hold substantial promise for overcoming therapeutic resistance, improving clinical outcomes, and enhancing quality of life for patients with GI cancers. Looking forward, advances in artificial intelligence-assisted drug discovery, precision oncology, and targeted protein degradation technologies are expected to accelerate the translation of E3 ligase biology into clinically effective therapies for gastrointestinal malignancies.
